# Terrestrial Macrofungal Diversity from the Tropical Dry Evergreen Biome of Southern India and Its Potential Role in Aerobiology

**DOI:** 10.1371/journal.pone.0169333

**Published:** 2017-01-10

**Authors:** Hema Priyamvada, M. Akila, Raj Kamal Singh, R. Ravikrishna, R. S. Verma, Ligy Philip, R. R. Marathe, L. K. Sahu, K. P. Sudheer, S. S. Gunthe

**Affiliations:** 1 EWRE Division, Department of Civil Engineering, Indian Institute of Technology Madras, Chennai, India; 2 Department of Chemical Engineering, Indian Institute of Technology Madras, Chennai, India; 3 Department of Biotechnology, Indian Institute of Technology Madras, Chennai, India; 4 Department of Management Studies, Indian Institute of Technology Madras, Chennai, India; 5 Physical Research Laboratory, Navarangpura, Ahmedabad, India; University of California Riverside, UNITED STATES

## Abstract

Macrofungi have long been investigated for various scientific purposes including their food and medicinal characteristics. Their role in aerobiology as a fraction of the primary biological aerosol particles (PBAPs), however, has been poorly studied. In this study, we present a source of macrofungi with two different but interdependent objectives: (i) to characterize the macrofungi from a tropical dry evergreen biome in southern India using advanced molecular techniques to enrich the database from this region, and (ii) to assess whether identified species of macrofungi are a potential source of atmospheric PBAPs. From the DNA analysis, we report the diversity of the terrestrial macrofungi from a tropical dry evergreen biome robustly supported by the statistical analyses for diversity conclusions. A total of 113 macrofungal species belonging to 54 genera and 23 families were recorded, with Basidiomycota and Ascomycota constituting 96% and 4% of the species, respectively. The highest species richness was found in the family *Agaricaceae* (25.3%) followed by *Polyporaceae* (15.3%) and *Marasmiaceae* (10.8%). The difference in the distribution of commonly observed macrofungal families over this location was compared with other locations in India (Karnataka, Kerala, Maharashtra, and West Bengal) using two statistical tests. The distributions of the terrestrial macrofungi were distinctly different in each ecosystem. We further attempted to demonstrate the potential role of terrestrial macrofungi as a source of PBAPs in ambient air. In our opinion, the findings from this ecosystem of India will enhance our understanding of the distribution, diversity, ecology, and biological prospects of terrestrial macrofungi as well as their potential to contribute to airborne fungal aerosols.

## Introduction

Fungi, one of the most important components of the ecosystem, comprise the largest biotic community after insects and include thousands of lineages, from the mushroom-forming fungi to yeasts, rusts, smuts, mold, and other symbionts with differing phenotypic and genotypic features [1]. Only 50% of the 1.5 million fungi present in the world have been identified and characterized thus far [1]. Macrofungi, which are visible to the naked eye (≥1 cm in size), possess mature spore-bearing and morphologically distinct fruiting bodies [2–4]. Of the four fungal phyla recognized, macrofungi belong to the Ascomycota (AMC) and Basidiomycota (BMC) [5]. Macrofungi (also called mushrooms) are represented by 41,000 species across the globe; however, only ~2% have been reported from India, despite the fact that one-third of the total global fungal diversity exists in the tropical Indian region [6]. Mushrooms, which naturally prefer all types of soil, grassy ground, rotten wood, leaf litter, decaying organic matter, etc., have the ability to grow in different seasons, yet all exhibit enhanced growth during the rainy season [7]. Macrofungi are important economically due to their importance in food, medicine, bio-control, chemical, biological and other industries [8]. Although the macrofungi are an integral part of a given ecosystem, their diversity and types are poorly studied, with a particular knowledge gap in the tropical regions including India [6,9]. Furthermore, macrofungi are not only significant in the terrestrial ecosystem but also play an important role in the atmospheric biogeochemical cycles by acting as a potential source of bioaerosols, mainly as fungal spores [5,10,11]. Fungal aerosols are portions of the fungal bodies that are small enough to become airborne and mostly involve the spores, hyphae and mycelia. Fungal spores comprise a large proportion of outdoor coarse particles (1–10 μm) released either actively or passively from their parent bodies [10, 12]. Ambient fungal aerosols originate mainly from the fungi growing on plant/tree surfaces or from the fungi thriving in the soil. Previous studies have reported that fungi can contribute 4–11% of the mass of fine particulate matter (PM_2.5_, particulate matter ≤ 2.5 μm) and 21% of the coarse particulate matter (PM_10_, particulate matter ≤ 10 μm) [10]. With a global emission rate of ~50 Tg yr^−1^, the number and mass concentrations of fungal spores in the continental boundary layer are of the order of 10^3^–10^7^ m^-3^ and ~1 μg m^-3^, respectively [13]. Interestingly, among all of the sources, nearly 34% (~17 Tg yr^−1^) is contributed by wet discharged macrofungal basidiospores [5]. Thus, macrofungi also play an important role in the atmospheric system by releasing fungal spores into the air. However, this aspect of macrofungi with its link to aerobiology has been largely overlooked. For example, mycologists have focused on investigating the diversity of terrestrial macrofungi [1,7,14,15]. On the other hand, the focus of aerobiologists has been mainly to characterize the types and diversity of airborne fungal spores [10,12,16–18]. Fungal spores can be transported over large distances by dispersion in air. Once these spores are in contact with the proper substrate under optimal conditions (e.g., with nutrients, moist conditions under high temperature, etc.), the growth of new fungi is initiated [19–21]. Because of their abundant and widespread dispersal in the atmosphere, these spores can adversely affect plants and animals, including humans [22–24]. It is now widely accepted that certain fungal spores are also capable of initiating the formation of ice nuclei in deep convective clouds at relatively warmer temperatures than required for homogeneous ice nucleation, thus affecting the hydrological cycle [25,26]. Therefore, considering the importance of macrofungi in the ecosystem, including their role in aerobiology, it is important to investigate their types, abundance, and diversity over various ecosystems including characterizing their sources. A large number of studies investigating the characteristics of macrofungi rely on traditional field-guide-based identifications, optical microscopy, and nutrient-specific culturing. However, the widely followed traditional morphologically based identifications have the following disadvantages: (i) the entire field-guide-based identification exercise is largely dependent on a significant expert or expertise needed for an unbiased identification [27]; (ii) many of the macrofungi may appear to have similar morphological features, and thus, identifications based on a field guide become difficult [28]; and (iii) identifications based mainly on the color and texture of the pileus, lamellae, and stipe (environmentally influenced traits) often differ from what is presented in the field guides. Hence, the biases involved in the identification become significant, and many of the macrofungi are often identified only to the family level [29]. Furthermore, this approach may not be helpful in providing the key information to establish the link between the release of macrofungal spores and their role in aerobiology. With DNA-based identification of macrofungi targeting the non-coding internal transcriber spacer region (ITS) of nuclear ribosomal rRNA, an unbiased and rapid identification of macrofungi down to the genus and species levels is possible [30], which can be further compared with a similar analysis carried out on airborne fungal spores [10] to establish the role of macrofungi as the source of airborne fungal spores. We present a first attempt to characterize the source of the terrestrial macrofungi in view of their potential role in contributing to atmospheric bioaerosols and regional aerobiology with the following objectives: (i) to assess the terrestrial macrofungal diversity and abundance in a rare tropical dry evergreen ecosystem, and (ii) to explore their potential in aerobiology by investigating the morphological characteristics of fungal spores, followed by modeling the spore dispersal in ambient air using the Gaussian plume model.

## Methods

### Study region

The tropical dry evergreen biome stretches along the eastern coast of India from northeastern to southeastern Tamilnadu, covering an area of ~25,500 km^2^. The study site is located in the coastal city of Chennai, which is within the tropical dry evergreen biome. Therefore, this study area is representative of the “tropical dry evergreen biome.” The climate of Chennai is typically hot and humid with three distinct meteorological seasons of summer (Feb–May), monsoon (Jun–Sep) and winter (Oct–Jan). Unlike the other major geographical part of India, which receives ~ 80% of the total annual rainfall during the southwest monsoon season (Jun–Sep), Chennai receives the majority of its rainfall during the northeast monsoon (Nov–Jan). Thus, the growth and persistence of macrofungi are expected to be higher over this region during the northeast monsoon. The sampling site, the Indian Institute of Technology Madras (IITM; 12.99°N, 80.23°E, 6 m amsl—above mean sea level), spreads across 687 acres, 18% of which is occupied by human establishments. The IITM is part of Guindy National Park, the 8^th^ smallest national park in India that occupies 1300 acres and is considered an “ecological island,” as it shelters native biodiversity within a mega-city [31]. The IITM campus is covered by a dense population of trees over the entire area with vast varieties of flora and fauna, the former consisting of 36% trees and 24% herbs, the rest being shrubs, climbers, grasses, and palm trees. The basic structure and floristic composition of the vegetation are similar to those of the “Tropical Dry Evergreen Forest” [31]. The campus consists of nearly 432 species of plants and animals together, with more than 300 different species of plants alone. Many groups of organisms such as bryophytes, fungi, spiders, insects, and butterflies are likely to extend the list of species on the campus given the favorable conditions for their growth and survival [31]. Thus, we are confident that the macrofungi sampled over this region and their characteristic features can be extrapolated to other similar ecosystems (especially the tropical dry evergreen biome) and the rest of the world where the investigated species are found. However, it must be noted that additional species not recorded in this study may well be present in the tropical dry evergreen biome of various other parts of the world.

### Macrofungal sampling

A detailed survey of the entire study region was conducted mainly to locate the regions where macrofungi are abundant. The following generic criteria for sampling the macrofungi based on the following two points were adopted: (i) sample collection based on the sporocarp, i.e., the fruiting body or fruit body; and (ii) sample collection based on the presence of mycelium and other microscopic parts including the hyphae [32,33]. For this study, the combination of both of the above methods, nominally referred as the ‘opportunistic sampling of macrofungi’ protocol, was followed [33]. This sampling method involves walking throughout the entire study region and collecting the macrofungi [33] covering up to 80% of the 687 acres, with the remaining 20% being deep wilderness with restricted human access. A total of 165 macrofungal samples were collected during the northeast monsoon and winter seasons (October–December 2014 and January and early February 2015). Sampling of the ground-dwelling macrofungi involved careful extraction of the fruit body from the soil and leaf litter, ensuring that the basal surface was intact. The tree-dwelling macrofungi were sampled by cutting off the basidiomata from the trees without disturbing the spore surfaces present below. Tree-dwelling macrofungi beyond the reach of manual sampling were not considered in the present study. All collected samples were carefully wrapped in aluminum foil, placed in an air-tight zip-lock bag and labeled with the collection number, location, date, and other data. The samples were preserved at -80°C (Forma 88000 Series -86°C, Thermo scientific, India) until further experimental analyses. For the samples collected at distances more than 1 km, a dedicated container with temperature control (~4°C) using an ice pack was used.

### Molecular biological analysis

#### DNA extraction

The DNA extraction was carried out using the commercially available Helini^™^ Purefast plant genomic DNA extraction kit (Helini ^™^ Biomolecules, India). Approximately 10–50 μg of flesh from the sporocarp was directly crushed using a plastic pestle in 1.5 ml Eppendorf ^®^ tubes, and the ensuing extraction steps were performed as per the supplier’s instructions. The extracted DNA was eluted in 50 μl of elution buffer provided in the kit and stored at -20°C until further use. The quality of the DNA was checked using agarose gel electrophoresis (1% gel; Bioworld^™^ electrophoresis setup, India).

#### Polymerase Chain Reaction (PCR)

The extracted DNA (0.5–1 μl) was used as a template for the PCR to obtain a sufficient quantity of DNA to be further used for sequencing. The rDNA ITS region was targeted for amplification using the ITS 4 (TCCTCCGCTTATTGATATGC) and ITS 5 (GGAAGTAAAAGTCGTAACAAGG) primers [34], which were obtained from Eurofins^®^, Bangalore, India. The master mix was prepared using 3 μl of 10× PCR buffer, 0.5 μl of 10 mM dNTP (Thermo Scientific^®^, catalog number: R0191) and 0.4 μl of 5 U of Taq DNA polymerase (Thermo Scientific^®^, catalog number: EP0402), with the total volume adjusted to 20 μl using nuclease-free water (NEB, catalog number: 12931S). To the 20 μl of master mix, 1 μl of 1 pmol/μl of each primer and 0.5–1 μl of DNA was added and the total volume was adjusted to 30 μl using nuclease-free water. The following sequence of thermal cycling operations was performed using the Surecycler 8800^®^ (Agilent Technologies): PCR with initial denaturation at 95°C for 5 minutes, followed by 35 cycles of denaturation at 95°C for 30 seconds, annealing at 55°C for 30 seconds, and elongation at 72°C for 30 seconds, with a final extension at 72°C for 3 minutes. The PCR products were then separated by electrophoresis (50 V to 100 V) on a 1.5% agarose gel and visualized using a gel documentation system (Gelstan, India) (S1 Fig).

#### Restriction Fragment Length Polymorphism (RFLP) and sequencing

The PCR products were purified using the PCR cleanup kit (Helini^™^ Purefast Gel/PCR cleanup kit), and RFLP analysis was performed to select different possible samples/species for sequencing. PCR amplicons were digested using three different restriction enzymes, viz., MspI (Thermo Scientific^®^, catalog number: ER0541), TaqI (Thermo Scientific^®^, catalog number: ER0671) and HinfI (Thermo Scientific^®^, catalog number: ER0801). The digestion mixture consisted of 5 μl of purified PCR product, 2 μl of 10 U restriction enzyme, and 3 μl of 10× RE buffer, and the reaction volume was adjusted to 30 μl using sterile Milli-Q water. The digestion was carried out for 1 h as per the supplier’s instructions, and bands were recorded to confirm the desired separation of species by electrophoresis on a 2–3% gel at 100 V (S2 Fig). The purified PCR products were then sent to three different sequencing facilities: (i) Xcelris labs, (Ahmedabad) India, (ii) Eurofins, (Bangalore) India, and (iii) Shrimpex, (Chennai) India. All of these service providers used the ABI 3730*xl* 96 capillary systems using the Big Dye Terminator v3.1 kit to obtain the sequences. The sequences for all 113 species have been deposited in the GenBank database, and the accession information obtained after depositing the sequences from NCBI is provided in Table A in S1 Text.

### Statistical analysis

The possibility of under-sampling bias in our study was addressed by performing various statistical analyses for the simulated sub-samples derived from bootstrapping (for 50 simulations). The obtained results are shown as a comparison between the ‘actual observations (obs)’ and ‘simulated/re-sampled observations (res)’. To quantify the relative abundance and diversity of the macrofungi in terms of ecological measures over the study region, different statistical indices were derived [35]. During our study, it was prominently noticed that fungi were either growing on trees (‘tree-dwelling’ fungi) or on the ground (‘ground-dwelling’ fungi). To find the diversity between these two fungi, Simpson’s dominance index (1-D), Shannon Weiner’s diversity index (H), and Shannon’s evenness of equitability (E_h_) were used. It is also important to note that certain species were not only found to grow in this ecological system but also in different ecosystems than the tropical dry evergreen biome [4,14,36–40]. To understand the differences in the distribution of species at the family level over different regions of India (Kerala, Karnataka, Maharashtra and West Bengal), we applied the Chi-square test and paired t-test. The families commonly and abundantly observed in different ecosystems reported previously and in this study were categorized into four major families (as discussed below). The families that were not commonly observed in all collective ecosystems were termed as “other families.” The details of the species reported for all of the compared regions mentioned above were obtained from the literature, and details regarding their sampling are presented as Table B in S1 Text. The Chi-square test was chosen as it is applicable to simple random samples and each categorical variable had an expected frequency count of at least 5. This enabled us to perform the Chi-square test and paired t-test to elucidate the differences in the distribution of the macrofungi that have been reported from different ecological systems in India. A p-value <0.05 was considered significant at a 95% confidence interval. To infer the differences among the macrofungal distributions on a one-on-one basis, a paired t-test was applied as discussed below. The paired t-test compares two population means where the observations in one sample can be paired with observations in the other sample. In our case, the compared families were the same, while the species belonging to the families *Agaricaceae*, *Marasmiaceae*, *Pluteaceae*, *Polyporaceae* and *‘other families’* were different. Thus, by applying these statistical techniques, we were able to identify the regions with higher distributional variability for the common families among them.

### Morphological characterization of the fungal spores

#### Extraction of fungal spores from the sporocarp

The morphological characteristics of the fungal spores were investigated using scanning electron microscopy (SEM) and fluorescence microscopy. Fungal spores were extracted manually from both fresh and mature sporocarps. A sterile scalpel was initially used to mechanically disturb the sporocarp to loosen the spores attached to the basidium by the sterigmata. The spores from the scalpel were carefully tapped onto a sterile glass slide in a drop (2–3 μl) of sterile nuclease-free water. The slide was air-dried prior to microscopic analysis by microscopy. Fluorescence microscopy and scanning electron microscopy were used to investigate the morphology of the fungal spores.

#### Fluorescence microscopy

To confirm the presence of spores and to understand their planar structure, fluorescence microscopy was performed prior to SEM. Fluorescence microscope images were obtained using the fluorescence NIKON Eclipse LV 100 (Japan) with an attached digital camera. A glass slide with the spore sample was placed in the specimen holder. Fungal spores were first visualized under bright-field microscopy at different magnifications ranging from 5× to 100×. In addition, spores were excited with an illumination source with excitation wavelengths of λex = blue (460–490 nm) and red (360–390 nm), and images were captured at emission wavelengths (λem) of 515–560 nm. After the fluorescence of the spores was confirmed, the slides were carefully stored at room temperature for SEM analysis.

#### Scanning Electron Microscopy (SEM)

The SEM (Quanta FEG 200, FEI) images of the fungal spores were obtained from the sophisticated analytical instrumentation facility (SAIF) located at IIT Madras. The central portion of the slide with the fungal spores was cut into a small square using a glasscutter pen. The slide was then sputtered with a thin layer of gold to render the surface conducive for obtaining secondary electron images to investigate the spores’ morphology.

### Macrofungal spore dispersion in the atmosphere

The behavior of spores in ambient air after their liberation and dispersal is of particular interest to scientists working in the field of fungal aerobiology to study their impact on climate and ecosystem health [41–43]. The role of macrofungi as a strong source of fungal spores in the atmosphere has been investigated using the Gaussian plume model (GPM) [44,45] for standard atmospheric conditions. Here, it is assumed that the source (a spore emission rate of 540 spores cm^-2^ s^-1^ [46]) is located 10 cm above the ground and that the spore concentrations were derived within a vicinity of 100 m and up to a height of 10 m (PM_10_ sampling was performed at a height of 10 m in the same location during the same study period; however, the PM_10_ results are not shown here).

To estimate the concentration, we set the following conditions: (i) the fruiting body height as 10 cm from the ground, (ii) an average spore size of 3 μm, (iii) an initial spore release rate of Q = 540 spores cm^-2^ s^-1^ [46], and (iv) an average wind speed of 1.79 m/s (the average wind speed observed during the sampling period). The following steps were conserved in the dispersal estimation: (i) estimating the settling velocity, V_s_, [45] (S1 Text); (ii) estimating the rate of convective airflow in terms of the gravity current, U_g_ [46]; and (iii) estimating the presence of spores in terms of their concentration for a fixed height (10 m) using GPM [44]. The settling velocity, V_s_, estimated for the average spores of 3 μm (the dominant size range of fungal spores observed on a global scale) [13,47–49], was incorporated in the gravity current estimations. For the gravity current estimations, we followed the steps reported by Dressaire et al., 2016 [46]. Furthermore, the spore dispersal estimations were derived from the ground level where the fungal spore source is assumed to be located. The downwind (x) and the crosswind (y) distances from the source center varied from 10 to 100 m. The gravity current, U_g_ (m/s), was estimated and incorporated into the mean horizontal wind speed, U, and thus, the additive wind speed U* (U* = U_g_ + U) was taken for the spore concentration (C) estimations under ambient conditions. Further details about the model can be found in Spijikerboer et al., 2002 [44].

## Results and Discussion

### Diversity and distribution

From the DNA sequencing performed for 165 terrestrial macrofungi, a total of 113 unique species were identified. The remaining 52 samples commonly belonged to 113 identified species. The identified species were further categorized into 54 different genera and 23 families. A comprehensive table describing all 113 identified species with other information such as family, distribution, characteristic properties, nominal distribution, and their presence as biological aerosols in the atmosphere based on previous literature is presented as Table A in S1 Text. In addition, brief descriptions of the 113 different identified species are presented in S1 Text.

The characteristics of the macrofungi predominantly observed during the rainy period (northeast monsoon) were distinct; they appeared to be fleshy and gilled and were mostly ground dwelling. During the wet periods, the high water content in the substrate and atmosphere, favorable temperature, and occasional sunlight favor the growth of fleshy and gilled macrofungi [7,50]. These types of macrofungi were generally observed to undergo a rapid growth at the end of the rainfall, while the presence of sufficient sunlight enhanced the availability of nutrients from the substrate [7]. Under these conditions, the larger macrofungi appeared to have a longer lifespan, whereas the smaller ones exhibited shorter lifespans. The pronounced variations in the moisture resulting from discrete or episodic rainfall events could cause the disappearance of smaller macrofungi [51]. Unlike ground-dwelling macrofungi, the tree-dwelling forms were consistently observed during rainy as well as non-rainy episodes, including dry periods. The ability of the large woody perennial polypores to survive both in the dry and moisture-rich periods could be attributed to their hard external upper fruiting body, which prevents water-loss during dry periods. Second, the vegetative mycelium that is deeply penetrated into the tree trunk obtains the water necessary for growth, survival and sustained spore release into the atmosphere. Third, the structurally significant, long and narrow hymenial tubes facilitate that the fungus remains in a relatively saturated atmosphere despite the dry environmental conditions [45]. Polypores belonging to the genera *Ganoderma*, *Trametes*, and *Coriolopsis* were present throughout the year in the study region, primarily for the aforementioned reasons.

All 113 terrestrial macrofungi identified to the species level were classified into two major phyla on the basis of DNA sequencing: (i) BMC comprised 96% of the total identified species and (ii) AMC comprised the remaining 4%. Basidiomycetes are predominantly gilled and fleshy fungi that appear immediately after a rainfall. Ascomycetes, the sac fungi, also have a tendency to appear after a rainfall, but unlike the basidiomycetes, their rate of appearance after a thunderstorm or rain is relatively lower compared to the BMC. The study region is a dense forest area with trees, leaf-litter and rich soil that provide the necessary substrate and developmental support for macrofungi, particularly the basidiomycetes. The fleshy and gilled fungi require ample water for growth, which is provided by the rain-wetted, moisture-laden leaf litter and tree trunks; the moisture from the saturated air is an additional requirement for their rapid growth [7]. Both of these growth factors were adequate over the study region, leading to the overwhelming growth and appearance of the fleshy gilled basidiomycetes that constituted the largest percentage (~96%). However, during the non-rainy seasons, these conditions may not be prevalent and the relative abundances between the BMC and AMC may vary considerably.

**Fig 1:**
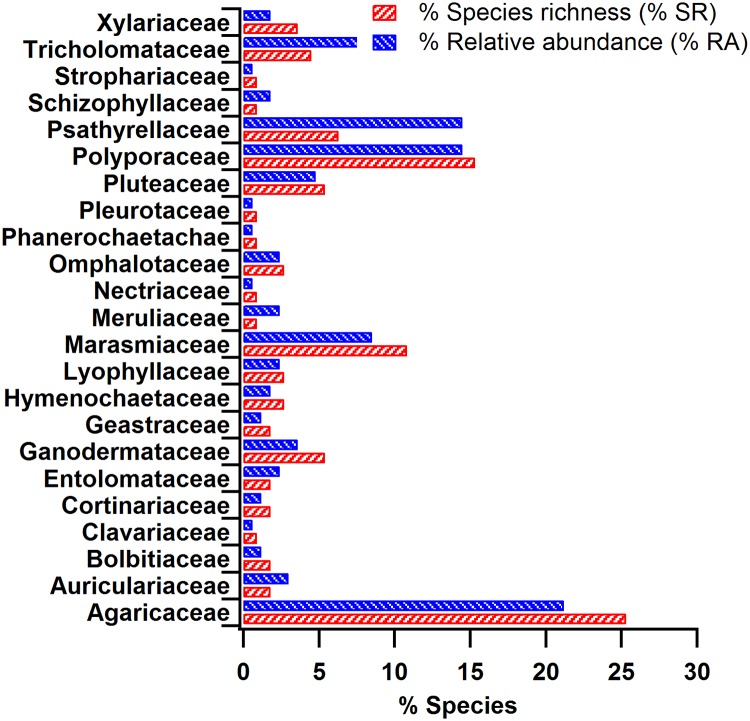
Species richness and abundance of all of the macrofungi identified to the genus level (113 species; 54 genera). Highest species richness was observed in the family *Agaricaceae* (25.3%) followed by *Polyporaceae* (15.31%) and *Marasmiaceae* (10.8%). *Agaricaceae* (21.2%) also had the highest % relative abundance followed by *Polyporaceae* and *Psathyrellaceae* with a contribution of 14.5% each.

The macrofungal diversity was further described in terms of species richness and % relative abundance [35]. In our study, *Agaricaceae* (25.3%) had the highest species richness, followed by *Polyporaceae* (15.3%) and *Marasmiaceae* (10.8%). Furthermore, the relative abundance of *Agaricaceae* (21.2%) was the highest, followed by *Polyporaceae* and *Psathyrellaceae* with contributions of 14.5% each. Details of the species richness and relative abundance of all of the observed species are depicted in Fig 1.

In addition to species richness, the relative dominance index up to the family level was derived using the Berger-Parker dominance index (‘d_obs_’–observed samples and ‘d_res_’–simulated/resampled samples). According to this index, the observed families were classified into three major categories: (i) dominant (D), (ii) general (G), and (iii) rare (R) [52]. The d values were reported to nominally range from 0 to 1. In the present case, the d values ranged from 0.006 to 0.212. Briefly, the family is considered to be predominantly present over a given region for a d value greater than 0.1. Similarly, d values between 0.01 and 0.1 are categorized as ‘generally’ present and d <0.01 indicates ‘rare’ occurrences of the family in a given region. Based on the ‘d_obs_’ values for the actual observed species, the *Agaricaceae*, *Polyporaceae* and *Psathyrellaceae* were the dominant families present over the study region, whereas families such as *Clavariaceae*, *Nectriaceae*, *Phanerochaetaceae*, *Pleurotaceae* and *Strophariaceae* were rare over the study region. In addition, a total of 15 families were in the general category in the overall macrofungi distribution (Table 1). The Berger-Parker dominance index obtained for the simulated/resampled (bootstrapping; d_res_) observations also indicated the same.

**Table 1:** % Species richness and relative abundance of various families over this study region calculated on the basis of the Berger-Parker dominance index. The dominance index was calculated for two categories: (i) for the samples observed (exact observations, n_obs_)–‘d_obs_’ and (ii) for the observations simulated 50 times incorporating the bootstrapping technique (resampled simulations, n_res_)–‘d_res_’. Based on this index, the observed families were classified into three major categories: (i) dominant (D), (ii) general (G), and (iii) rare (R). The dominant, general and rare species differed significantly for the observed species, whereas all of the simulated observations of the macrofungal species were general in the study region. Please refer to the main text for more details related to the methodology of obtaining the relative dominance index.

S.No	Family	n_obs_	‘d_obs_'	Relative dominance	n_res_	‘d_res_’	Relative dominance
1	*Agaricaceae*	35	0.212	D	28	0.217	D
2	*Auriculariaceae*	5	0.03	G	4	0.028	G
3	*Bolbitiaceae*	2	0.012	G	2	0.014	G
4	*Clavariaceae*	1	0.006	R	1	0.005	R
5	*Cortinariaceae*	2	0.012	G	2	0.012	G
6	*Entolomataceae*	4	0.024	G	3	0.026	G
7	*Ganodermataceae*	6	0.036	G	5	0.037	G
8	*Geastraceae*	2	0.012	G	2	0.014	G
9	*Hymenochaetaceae*	3	0.018	G	2	0.016	G
10	*Lyophyllaceae*	4	0.024	G	3	0.021	G
11	*Marasmiaceae*	14	0.085	G	10	0.075	G
12	*Meruliaecea*	4	0.024	G	4	0.029	G
13	*Nectriaceae*	1	0.006	R	1	0.007	R
14	*Omphalotaceae*	4	0.024	G	3	0.02	G
15	*Phanerochaetachae*	1	0.006	R	1	0.008	R
16	*Pleurotaceae*	1	0.006	R	2	0.018	R
17	*Pluteaceae*	8	0.048	G	6	0.047	G
18	*Polyporaceae*	24	0.145	D	18	0.141	D
19	*Psathyrellaceae*	24	0.145	D	18	0.141	D
20	*Schizophyllaceae*	3	0.018	G	3	0.02	G
21	*Strophariaceae*	1	0.006	R	1	0.007	R
22	*Tricholomataceae*	13	0.079	G	10	0.076	G
23	*Xylariaceae*	3	0.018	G	2	0.016	G

### Statistical analysis

The diversity and distribution of the macrofungi over the study region were quantified using the statistical techniques described in section 2.4; the inferences made are discussed below.

#### Diversity index

The diversity indexes for the ground-dwelling (‘GD_obs_’–observed samples and ‘GD_res_’–simulated/resampled species) and tree-dwelling (‘TD_obs_’–observed samples and ‘TD_res_’–simulated/resampled species) macrofungi were derived using Shannon’s diversity index (H), Gini-Simpson’s diversity index (1-D), and Shannon’s evenness of equitability (E_H_) index; a summary of these indices is listed in Table 2. Based on derived indices, we infer that tree-dwelling species were more diverse compared to ground-dwelling macrofungi for both the observed and simulated/resampled species. Furthermore, the E_h_ values indicated that tree-dwelling macrofungi were more evenly distributed compared to the ground-dwelling macrofungi. Gini-Simpson’s diversity index was higher (TD_obs_ = 0.97) for the tree-dwelling macrofungal community compared to the ground-dwelling macrofungal community. A higher Gini-Simpson’s diversity index value indicates a higher diversity of species in the study region. Thus, the tree-dwelling macrofungal community had a higher diversity than the ground-dwelling macrofungal community throughout the study region. Shannon’s diversity index (H) of the tree-dwelling macrofungal community (TD_obs_ = 3.22) was also higher than that of the ground-dwelling species, indicating a higher diversity of the tree-dwelling macrofungi community. The higher value of species evenness (E_H_) for the tree-dwelling macrofungal community (TD_obs_ = 0.823) indicated a more even distribution of the tree-dwelling macrofungi in the study region.

**Table 2:** Diversity indices calculated for the ground-dwelling and tree-dwelling macrofungi. Tree-dwelling species were found to be more diverse and even in both the categories of ‘observed’ and ‘simulated/resampled (res)’ data.

Indices	GD_obs_	GD_res_	TD_obs_	TD_res_
Simpson's Diversity index '1-D'	0.959	0.978	0.97	0.986
Shannon's Diversity index 'H'	3.13	3.36	3.22	3.39
Evenness 'E_h_'	0.737	0.787	0.823	0.871

#### Distribution of macrofungi in different regions of India

**Fig 2:**
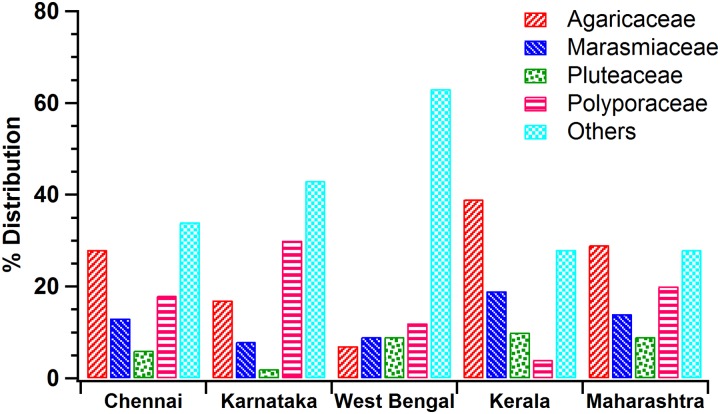
Distribution of macrofungi in different regions of India belonging to the families Agaricaceae, Marasmiaceae, Pluteaceae, Polyporaceae and ‘other families’. Chennai was compared with Karnataka, West Bengal, Kerala and Maharashtra.

The differences in the distribution of the families *Agaricaceae*, *Marasmiaceae*, *Pluteaceae*, *Polyporaceae* and other families for different regions of India were statistically analyzed using the Chi-square test and paired t-test. Fig 2 shows the macrofungal spread over the study region (Chennai), Karnataka, West Bengal, Kerala, and Maharashtra.

To determine significant differences in the distribution of common macrofungal families in five compared regions, namely Chennai (the study region), Kerala, Karnataka, West Bengal, and Maharashtra, the Chi-square test was performed. From the p-value <0.05, it was evident that the macrofungal distribution varied in different regions of India. Table C in S1 Text shows the observed and expected frequencies of the Chi-square test performed for the actual observations and the simulated/resampled observations. A paired t-test was also performed to find the regions with greater/similar macrofungal distributions. From the values, p = 0.04 < 0.05 and p = 0.03 < 0.05, it can be seen that Karnataka and Kerala had greater distributions of macrofungi compared to Chennai for the families that were common between them. The values, p = 0.06 > 0.05 and p = 0.29 > 0.05, indicated that Chennai had a macrofungal distribution that was similar to West Bengal and Maharashtra. The paired t-test performed for the simulated/resampled observations yielded the same conclusions as shown in Table 3.

**Table 3:** Paired t-test performed for the actual and simulated observations. The p-values for the pairs varied between the actual and the simulated observations; however, the conclusions drawn remained the same.

p—value	Chennai Vs. Karnataka	Chennai Vs. West Bengal	Chennai Vs. Kerala	Chennai Vs. Maharashtra
**Hypothesis**	H_0_: μ_Ka_ - μ_Ch_ = 0, H_a_: μ_Ka_ - μ_Ch_>0	H_0_: μ_Ch_ - μ_WB_ = 0, H_a_: μ_Ch_ - μ_WB_>0	H_0_: μ_Ke_ - μ_Ch_ = 0, H_a_: μ_Ke_ - μ_Ch_>0	H_0_: μ_Ma_ - μ_Ch_ = 0, H_a_: μ_Ma_ - μ_Ch_>0
**Observed**	0.04 < 0.05	0.06 > 0.05	0.03 < 0.05	0.29 > 0.05
**Conclusion**	Karnataka had a greater distribution	Chennai and West Bengal had a similar distribution	Kerala had a greater distribution	Chennai and Maharashtra had a similar distribution
**Resampled**	0.04 < 0.05	0.06 > 0.05	0.02 < 0.05	0.22 > 0.05
**Conclusion**	Karnataka had a greater distribution	Chennai and West Bengal have a similar distribution	Kerala had a greater distribution	Chennai and Maharashtra had a similar distribution

### Morphological characterization of the macrofungal spores

**Fig 3:**
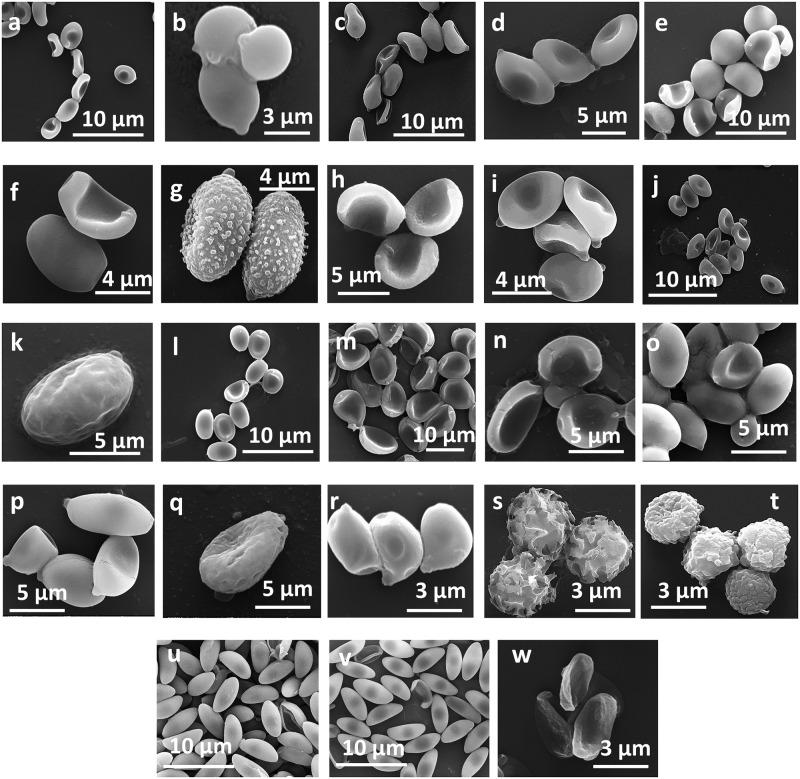
Scanning electron microscopic images of some the selected macrofungal species belonging to Ascomycota and Basidiomycota. a*- Agaricus hondensis*, b—*Agaricus moelleri*, c—*Chlorophyllum nothorachodes*, d—*Conocybe mandschurica*, e—*Coprinellus aureogranulatus*, f *-Coprinellus radians*,g—*Gymnopilus purpureosquamulosus*, h—*Hymenagaricus taiwanensis*,i—*Leucoagaricus atrodisca*, j—*Micropsalliota globocystis*, k—*Pholiota spumosa*, l—*Psathyrella candolleana (young spores)*,m—*Psathyrella candolleana (mature spores)*, n—*Psathyrella gracilis*, o—*Volvariella taylorii*, p—*Ceriporia lacerate*, q—*Ganoderma lucidum*, r—*Phellinus repandus*, s—*Geastrum pectinatum*, t—*Geastrum striatum*, u—*Daldinia eschscholzii*, v—*Cosmospora viliuscula and* w—*Xylaria cirrata*. Spores were observed in varying size and shapes. Scale is varying for each panel and is shown respectively.

**Fig 4:**
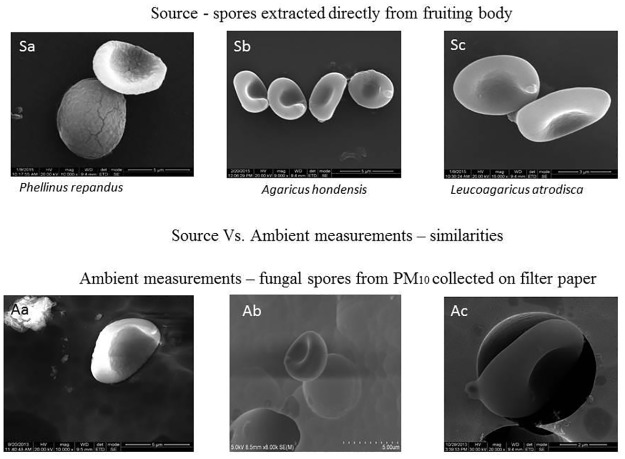
Source vs. Ambient spore morphological similarities. Panel of figures (Sa–Sc) at the top show manually extracted fungal spores identified to the species level. Panel of figures (Aa–Ac) at the bottom show the fungal spores collected from the ambient air on a polycarbonate filter paper as reported by Valsan et al., 2015 [41]. It is to be noted that the ambient fungal spores reported and depicted here were mainly observed during the NE monsoon season, the season with the fungal bloom. Both of the studies are from the same study region, IIT Madras and from same season of October–January (monsoon and winter in southern India, Chennai). Kindly refer Valsan et al., 2015 [41] for further details regarding the bioaerosols SEM study.

The primary objective of morphologically characterizing the fungal spores is to document the SEM images, which could be useful for further investigation and identification of the fungal spores in the atmosphere. As the fungal spores constitute a major fraction of ambient bioaerosols, the images from our study would assist in their efficient identification. A recent study in the Indian region by Valsan et al., 2015 [41] emphasized the use and importance of SEM techniques in studying the morphological features of fungal spores. The fluorescence microscopy images are presented in S3 Fig and the high-resolution SEM images in Fig 3. Note that our SEM images could be obtained for only 23 of the 113 species (Table D in S1 Text) due to the difficulty of extracting the fungal spores from the collected samples. For example, some of the macrofungi were too small and delicate to extract the spores without damaging them; if the spores were extracted at all, they turned out to be damaged and broken (S4 Fig). The shapes of fungal spores observed by SEM were predominantly ellipsoidal with a conspicuous apiculus, and few of the spores were globose, ovoid, or spherical. Generally, their sizes ranged from 3 μm to 12 μm, translating into equivalent aerodynamic diameters of ~ 2–6 μm (kindly refer to Table D in S1 Text for more details). Interestingly, few of the fungal spores characterized in this study at the source level were reported in the ambient atmosphere by Valsan et al. [41], who sampled the ambient air to characterize the morphology of atmospheric bioaerosols (Fig 4) at the same location. This again emphasizes the fact that terrestrial macrofungi could constitute a strong and potential source of atmospheric PBAPs. Moreover, to the best of our knowledge, many of the images presented here are the first that represent the morphological features of terrestrial macrofungi spores native to the tropical dry evergreen biome.

### The implication of the sources on aerobiology

The simultaneous characterization of macrofungi and atmospheric fungal spores would help in understanding the role and contribution of terrestrial fungi to atmospheric bioaerosols. This could be particularly important for the field of pathogenic aerobiology, as the presence of human, plant and animal pathogens in the air is well established [22,23]. Under this scenario, it is important to qualitatively and quantitatively investigate the link between the source of the fungal spores and their presence in the atmosphere. The quantitative estimation of fungal spores in the atmosphere is reported as spores/m^3^ by performing the GPM. Note, however, that these details are provided merely to explain the fact that spores emitted by the macrofungi are capable of contributing to the atmospheric bioaerosol burden through various physical mechanisms. Hence, these details are limited to our understanding and assumptions related to this particular example and should not be extrapolated beyond this particular study. The aerobiological pathway of the PBAPs involves the following steps: (i) emission from the source, (ii) takeoff/ascent, (iii) the actual flight, (iv) landing/descent, and (v) impact at the receptor [45]. Furthermore, the actual flight will determine the ability of the spore to act as a PBAP after its release by the host organism.

One of the most important characteristics of the spore from the point of view of dispersal is the retention of its capability to germinate. The effectiveness of the germination power of the spore is important for its role as a bioaerosol in an ecosystem’s health and climate. The viability of the fungal spores in the atmosphere depends on two important factors: (i) morphology—shape, size and predominantly pigmentation, and (ii) meteorological factors—relative humidity and radiation [53]. Many spores often die because of desiccation and the injurious effects of solar radiation [54]. Thin-walled spores seem to be particularly susceptible, while those with thicker walls are resistant to drying. Spores with pigmented walls have been reported to be more resistant to radiation. Thus, the thick-walled and pigmented spores have a high capability of remaining in the atmosphere for a longer time. In our study, we found that spores belonging to the families *Agaricaceae*, *Psathyrellaceae*, *Polyporaceae* and *Hymenochaetaceae* can remain alive for a relatively longer time during their flight as a result of their thick walls (S1 Text). Spores belonging to the families *Agaricaceae*, *Entolomataceae*, *Psathyrellaceae*, *Cortinariaceae*, *Strophariaceae*, *Ganodermataceae* and *Xylariaceae* are pigmented (S1 Text) and thus can withstand the effects of radiation. However, this preliminary information regarding the viability of the spores must be tested further with specific laboratory experiments. In our study, we found several pathogenic fungi that can affect plants (plant pathogens 72%), human pathogens causing respiratory allergies (human pathogens 7%), and poisonous fungi (21%) mainly belonging to Basidiomycota. The pathogenicity observed at the source level may have similarities with the pathogenicity of the fungal aerosols as the terrestrial macrofungi have an outstanding feature of producing enormous quantities of spores.

#### Dispersal of macrofungal spores in the atmosphere

**Fig 5:**
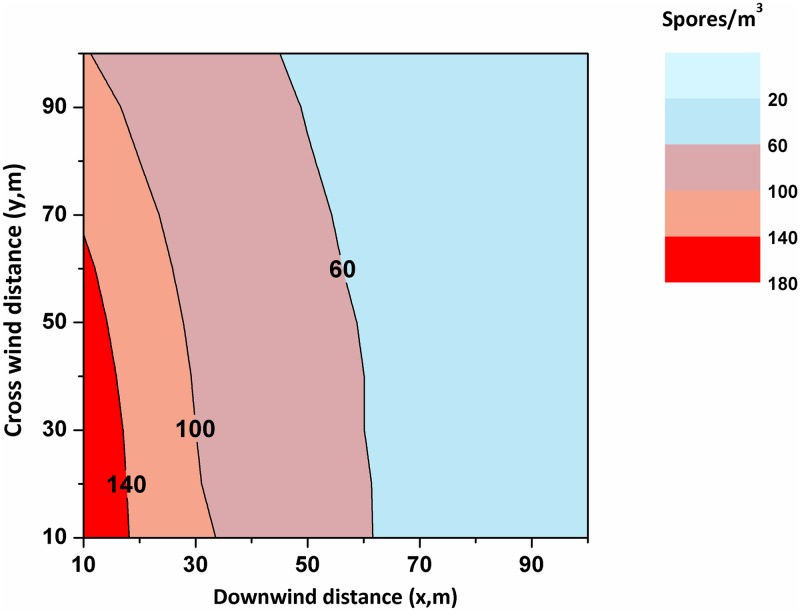
Fungal spore presence and the concentration distribution predicted at a height of 10m from the source using the Gaussian plume model. Spore concentrations were highest near the source. Gradients are also large near the source but gradually become less steep with the increasing downwind distance (x, m). Color shading indicates the spores/m3 of air for a stability class A. Release (C_initial_) was 540 × 10^4^ spores/m^2^/s and the ambient wind speed was 1.79 m/s; Presence of spores could also be seen at a location of (100, 100, 10) m from the source.

To show the potential role of macrofungi as a strong source of fungal spores in the atmosphere, we used the Gaussian plume model (GPM) [44] to estimate the spore concentrations for standard atmospheric conditions (and assuming an initial spore release rate of 540 spores cm^-2^ s^-1^ [46]). From the GPM, we were able to derive the concentration of spores/m^3^ at a height of 10 m (the z component) and their concentrations at various downwind (x in meters) and crosswind (y in meters) distances as shown in Fig 5 (Table E in S1 Text shows the calculated spore terminal velocity and fall speed). The modeling calculations revealed the presence of spores at even the farthest distances (x = 100, y = 100, and z = 10 meters) in the respective directions, and the corresponding estimated concentration was 35 spores/m^3^ of air. It should be noted, however, that the concentrations of spores were calculated assuming an instantaneous release (1 second). In reality, the release from the fruiting body occurs over longer time periods of seconds to hours [5] at regular intervals.

It is equally important to analyze the effect of the source height in the estimation of spore concentrations. In this regard, the generated gravity currents, U_g_, remain unchanged for all of the macrofungi regardless of their height from the ground, as the gravity current generation is valid up to a maximum height of 10–12 mm [46]. We observed that the wind velocity is greater than the gravity current (U_g_ >> U); hence, the source height alone may not be the deciding factor for spore dispersion under given ambient conditions. Thus, we can conclude that once the spores are released from the macrofungi, they can remain suspended in the air and can travel great distances. Norros et al. [55,56] also reported similar results that macrofungi have a very high potential of spore dispersal and that even under stable atmospheric conditions, up to 95% of the spores released can disperse beyond 1 km.

#### Influence of rainfall on fungal spore release and air spore concentrations during the NE monsoon

**Fig 6:**
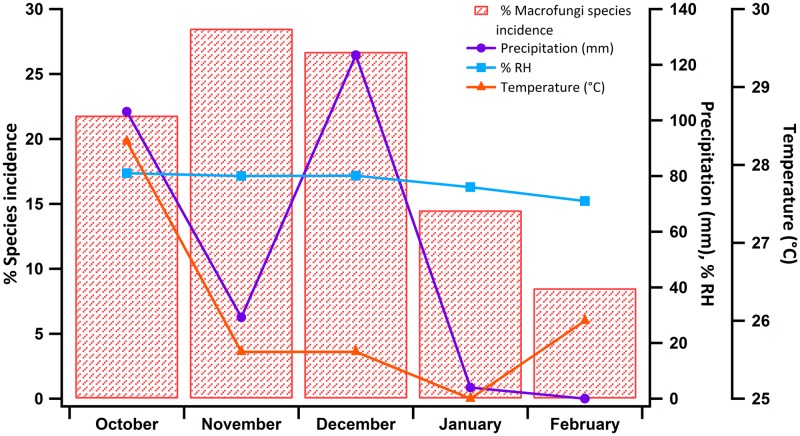
Incidence (%) of macrofungal species incidence during the sampling period (Oct–Feb 2015) comprising the winter season with rainfall (Oct–Jan 2015) and summer (Feb 2015). Macrofungal incidence is correlated with cumulative rainfall (mm) that occurred, % relative humidity (RH) and the temperature (°C). Incidence of macrofungi was found to be the maximum during November; the rainfall of October and highest % RH (80%) seemed conducive for the increased incidence of macrofungi during November. Again the rainfall during December further supported its growth and persistence throughout the months December and January. The decrease in the macrofungi incidence from December to February can be associated with the decrease in rainfall and increase in temperature.

**Fig 7:**
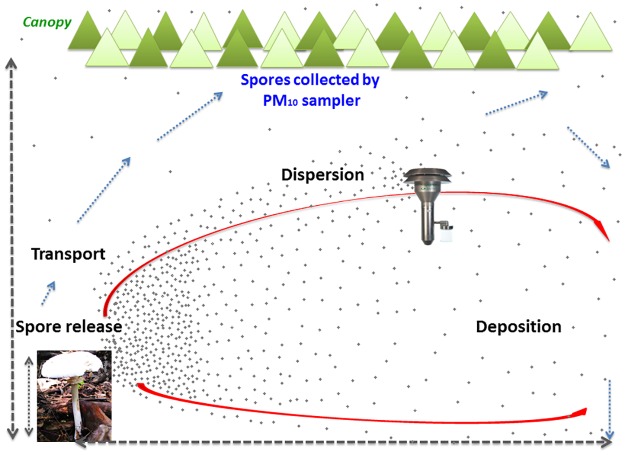
Conceptual spore movement framework in the study region. Macrofungi source is at a height of 10m from the ground. Spore release and dispersal viewed at a height (z) of 10 m, downwind distance (x) of 10–100 m and at a crosswind distance (y) of 10 to 100 m (crosswind not depicted). Spores released through convective currents had an U_g_ of 0.06 m/s and the wind velocity that existed (ambient) was U = 1.79 m/s. Spores released from the macrofungi can act as a potential bioaerosol as proven by GPM. DNA analysis of PM_10_ measurements done during the same period (not a part of this study), confirmed the presence of *Agaricaceae* and *Polyporaceae* found at the source.

The macrofungal blooms and sampling occurred mainly during the NE monsoon, the period of rainfall over the study region. We have already discussed how rain and moisture help or serve as a catalyst for fungal growth and persistence. Rain can also help in fungal spore dispersal from the macrofungi by means of mechanical splashing. On this basis, we hypothesize that for our study, rainfall has two major implications: (i) it provides enough moisture to support the growth and spread of macrofungi in the study region (Fig 6) and (ii) the splash-induced spore release from the fruiting body leads to the rise in the spore concentration in PM_10_ during rainy days (not discussed here). From Fig 6, the influence of rainfall on macrofungal growth is apparent over the study region. The months of November and December had the highest levels of macrofungi when the most conducive conditions of greatest rainfall and lowest temperatures existed. A summary of the meteorological conditions that existed during the sampling period is presented as Table F in S1 Text. As mentioned earlier, the PM_10_ characterization performed during the same period also revealed the increased presence of macrofungal spores in the filter samples with an overall contribution of 17% to the atmospheric PM_10_ (follow-up studies). Thus, it is evident how the seasonally occurring terrestrial macrofungi can contribute to atmospheric aerosols, and in this study, we attempted to establish the potential role of macrofungi as a strong source of fungal spores in the atmosphere (Fig 7).

## Conclusion

We present the types and diversity of the macrofungi from a rare tropical dry evergreen biome to the species level using advanced DNA analytical techniques. Out of 165 samples investigated, 113 different species were identified, which can potentially serve as baseline information for terrestrial fungal diversity of a tropical dry evergreen ecosystem. Basidiomycota was the dominant phylum, followed by Ascomycota in this ecosystem due to the favorable environmental conditions (moisture, rainfall, temperature, etc.) for the growth of basidiomycetes. From the derived indices, we demonstrated that tree-dwelling macrofungi were diverse and abundant over this region. The types of identified species were further subjected to statistical tests, and we found substantial differences in the distribution of families such as *Agaricaceae*, *Marasmiaceae*, *Pluteaceae*, and *Polyporaceae*. We also present the morphological characteristics of the fungal spores for 23 different macrofungal species using SEM and fluorescence microscopy. Their distinct morphological characteristics coincided with the typical morphological characteristics of the fungal spores found in the ambient atmosphere. The ability of macrofungi to travel greater distances in ambient conditions and thus behave as a potential bioaerosol was confirmed by the Gaussian plume modeling results.

Until now, our understanding of the biodiversity related to the macrofungi from this part of the world has been based on very limited scientific findings that lacked adequate sequencing data, detailed morphological characterization of the spores with reference to their role in aerobiology, and robust statistical description in terms of abundance and diversity. The data characterizing the macrofungi as a potential source of atmospheric bioaerosols are very limited for India. This opens up a need for more detailed studies over larger spatiotemporal scale. In view of these gap areas, we believe that this study will be an important contribution to the fields of plant pathology, epidemiology, aerobiology, and biomechanics, particularly over the Indian region.

## Supporting Information

S1 Fig PCR gel images of the amplified macrofungal DNA. Exemplary image of gel electrophoresis showing amplification of rDNA ITS of initially collected macrofungi sporocarp during the month of October. Lane 1: negative control; Lane 2: DNA from sample 1 which was found to be *Agaricus bohusii* after performing the sequencing; Lane 3: DNA from sample 2 which was found to be *Agaricus hondensis* after performing the sequencing; Lane 4: DNA from sample 3 which was found to be *Chlorophyllum globosum* after performing the sequencing; Lane 5: DNA from sample 4 which was found to be *Clitopilus giovanellae* after performing the sequencing; Lane 6: 100 bp plus DNA ladder; Lane 7: DNA from sample 5 which was found to be *Ganoderma lucidum* after performing the sequencing; Lane 8: DNA from sample 6 which was found to be *Lenzites elegans* after performing the sequencing; Lane 9: DNA from sample 7 which was found to be *Lepista nuda* after performing the sequencing; Lane 10: DNA from sample 8 which was found to be *Psathyrella candolleana* after performing the sequencing.
(TIF)

S2 Fig Exemplary Agarose Gel Electrophoresis DNA bands obtained using Restriction Fragment Length Polymorphism (RFLP). (a) RFLP gel image for amplified ITS region of *Agaricus bohusii*, *Agaricus hondensis*, *Chlorophyllum globosum* and *Clitopilus giovanellae* using the restriction enzyme *Hinf I* and (b) same as (a) but digestion was performed using the enzymes *MspI* and *TaqI*.
(TIF)

S3 Fig Brightfield and fluorescence microscopic images of fungal spores obtained using fluorescence microscope (Nikon Eclipse LV100). Particles were excited with an illumination source having an excitation wavelength of λex = Blue (460–490 nm) and Red (360–390 nm) and emission wavelength λem for the captured images was between 510–560 nm. The spores belonged to the following species: a—*Agaricus hondensis*, b*- Agaricus moelleri*, c—*Chlorophyllum nothorachodes*, d—*Conocybe mandschurica*, e—*Coprinellus aureogranulatus*, f *-Coprinellus radians*,g—*Gymnopilus purpureosquamulosus*, h—*Hymenagaricus taiwanensis*,i*- Leucoagaricus atrodisca*, j—*Micropsalliota globocystis*, k—*Pholiota spumosa*, l—*Psathyrella candolleana (young spores)*,m—*Psathyrella candolleana (mature spores)*, n—*Psathyrella gracilis*, o—*Volvariella taylorii*, p—*Ceriporia lacerate*, q—*Ganoderma lucidum*, r—*Phellinus repandus*, s—*Geastrum pectinatum*, t—*Geastrum striatum*, u—*Daldinia eschscholzii*, v *-Cosmospora viliuscula and* w—*Xylaria cirrata*.
(TIF)

S4 Fig Scanning electron microscopic images of exemplary damaged fungal spores. a *-Daldinia eschscholzii*, b—*Coprinellus aureogranulatus* and c *-Psathyrella gracilis*.
(TIF)

S1 Text Supplementary information. Contains tables (A to F), terminal velocity calculation, fungal spore fall speed calculation and an overview of the macrofungi species observed.
(DOCX)
