# Supplementary figures and images for: Terrestrial Macrofungal Diversity from the Tropical Dry Evergreen Biome of Southern India and Its Potential Role in Aerobiology
Source: PLoS One. 2017 Jan 10;12(1):e0169333. doi: 10.1371/journal.pone.0169333 (PMC5224982; doi:10.1371/journal.pone.0169333)

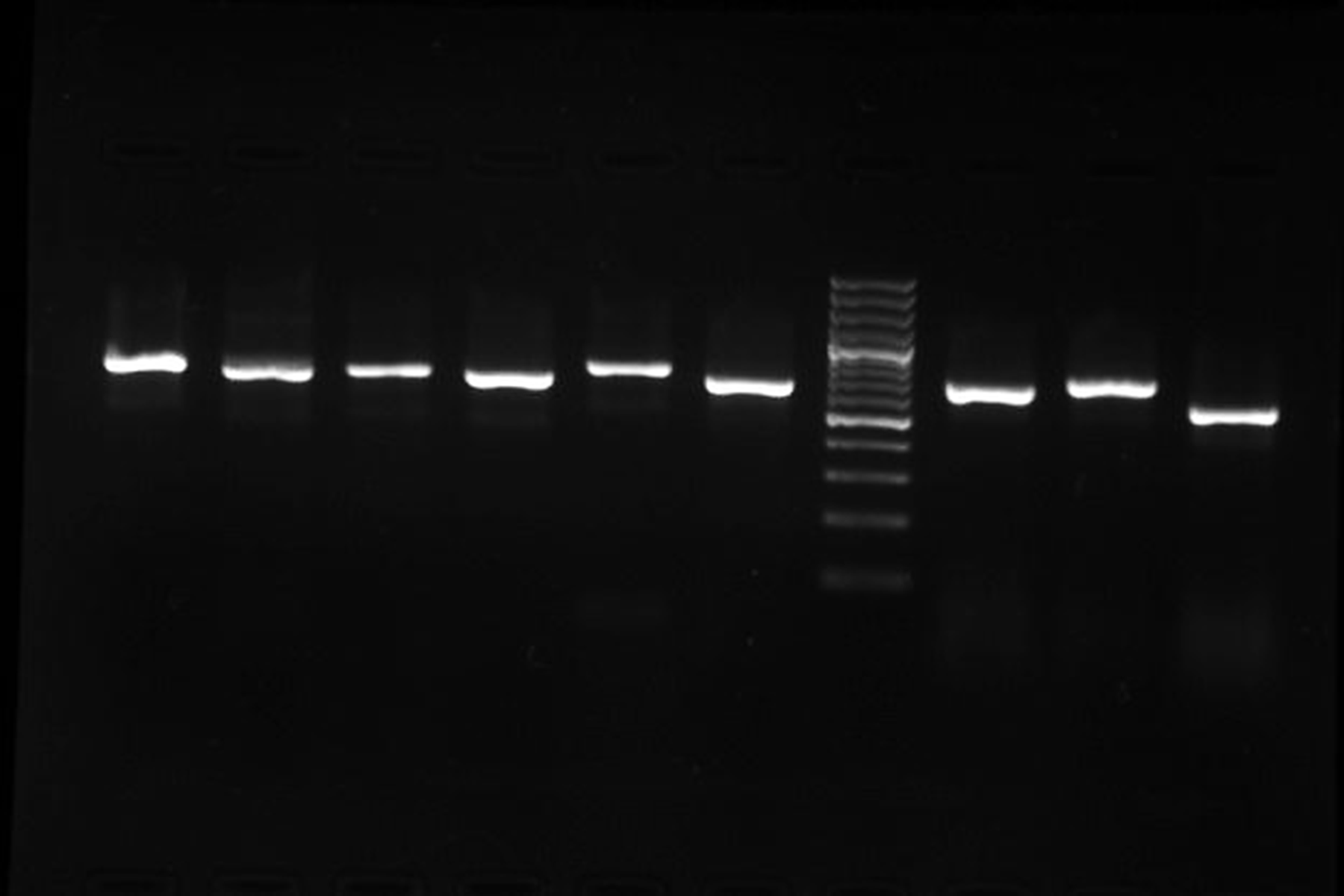

Supplement: S1 Fig — Exemplary image of gel electrophoresis showing amplification of rDNA ITS of initially collected macrofungi sporocarp during the month of October. Lane 1: negative control; Lane 2: DNA from sample 1 which was found to be Agaricus bohusii after performing the sequencing; Lane 3: DNA from sample 2 which was found to be Agaricus hondensis after performing the sequencing; Lane 4: DNA from sample 3 which was found to be Chlorophyllum globosum after performing the sequencing; Lane 5: DNA from sample 4 which was found to be Clitopilus giovanellae after performing the sequencing; Lane 6: 100 bp plus DNA ladder; Lane 7: DNA from sample 5 which was found to be Ganoderma lucidum after performing the sequencing; Lane 8: DNA from sample 6 which was found to be Lenzites elegans after performing the sequencing; Lane 9: DNA from sample 7 which was found to be Lepista nuda after performing the sequencing; Lane 10: DNA from sample 8 which was found to be Psathyrella candolleana after performing the sequencing. (TIF) [file pone.0169333.s001.tif]

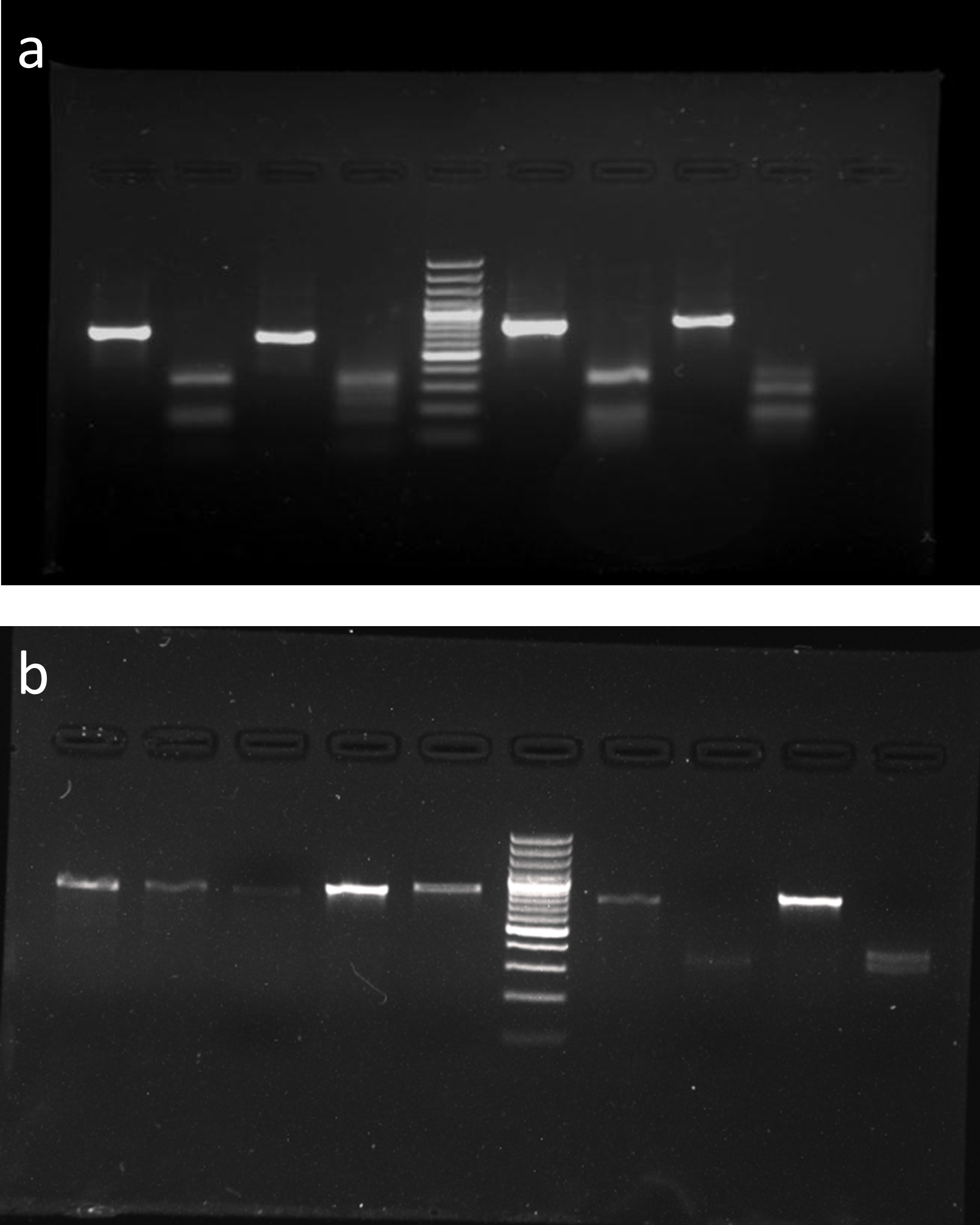

Supplement: S2 Fig — (a) RFLP gel image for amplified ITS region of Agaricus bohusii, Agaricus hondensis, Chlorophyllum globosum and Clitopilus giovanellae using the restriction enzyme Hinf I and (b) same as (a) but digestion was performed using the enzymes MspI and TaqI. (TIF) [file pone.0169333.s002.tif]

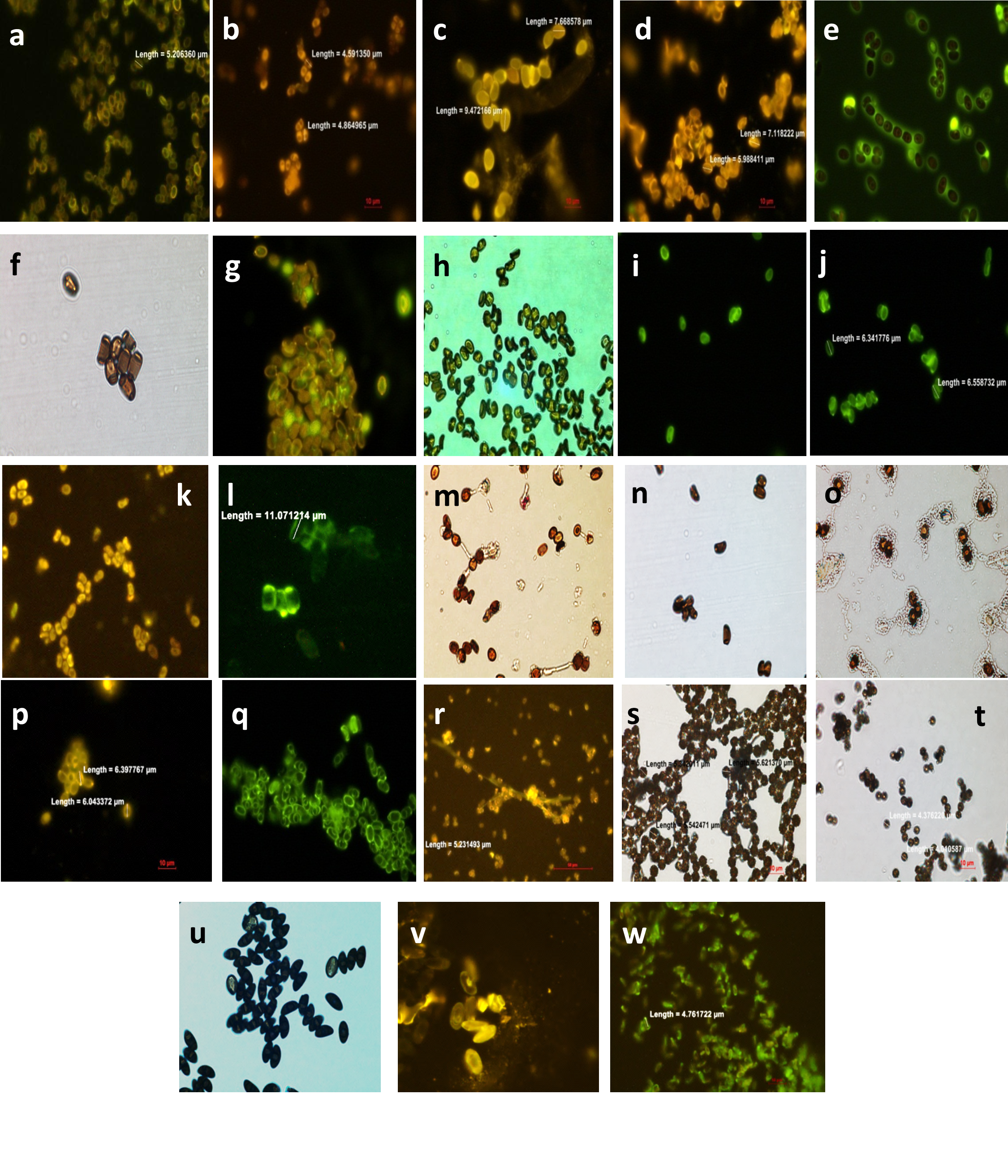

Supplement: S3 Fig — Particles were excited with an illumination source having an excitation wavelength of λex = Blue (460–490 nm) and Red (360–390 nm) and emission wavelength λem for the captured images was between 510–560 nm. The spores belonged to the following species: a—Agaricus hondensis, b- Agaricus moelleri, c—Chlorophyllum nothorachodes, d—Conocybe mandschurica, e—Coprinellus aureogranulatus, f -Coprinellus radians,g—Gymnopilus purpureosquamulosus, h—Hymenagaricus taiwanensis,i- Leucoagaricus atrodisca, j—Micropsalliota globocystis, k—Pholiota spumosa, l—Psathyrella candolleana (young spores),m—Psathyrella candolleana (mature spores), n—Psathyrella gracilis, o—Volvariella taylorii, p—Ceriporia lacerate, q—Ganoderma lucidum, r—Phellinus repandus, s—Geastrum pectinatum, t—Geastrum striatum, u—Daldinia eschscholzii, v -Cosmospora viliuscula and w—Xylaria cirrata. (TIF) [file pone.0169333.s003.tif]

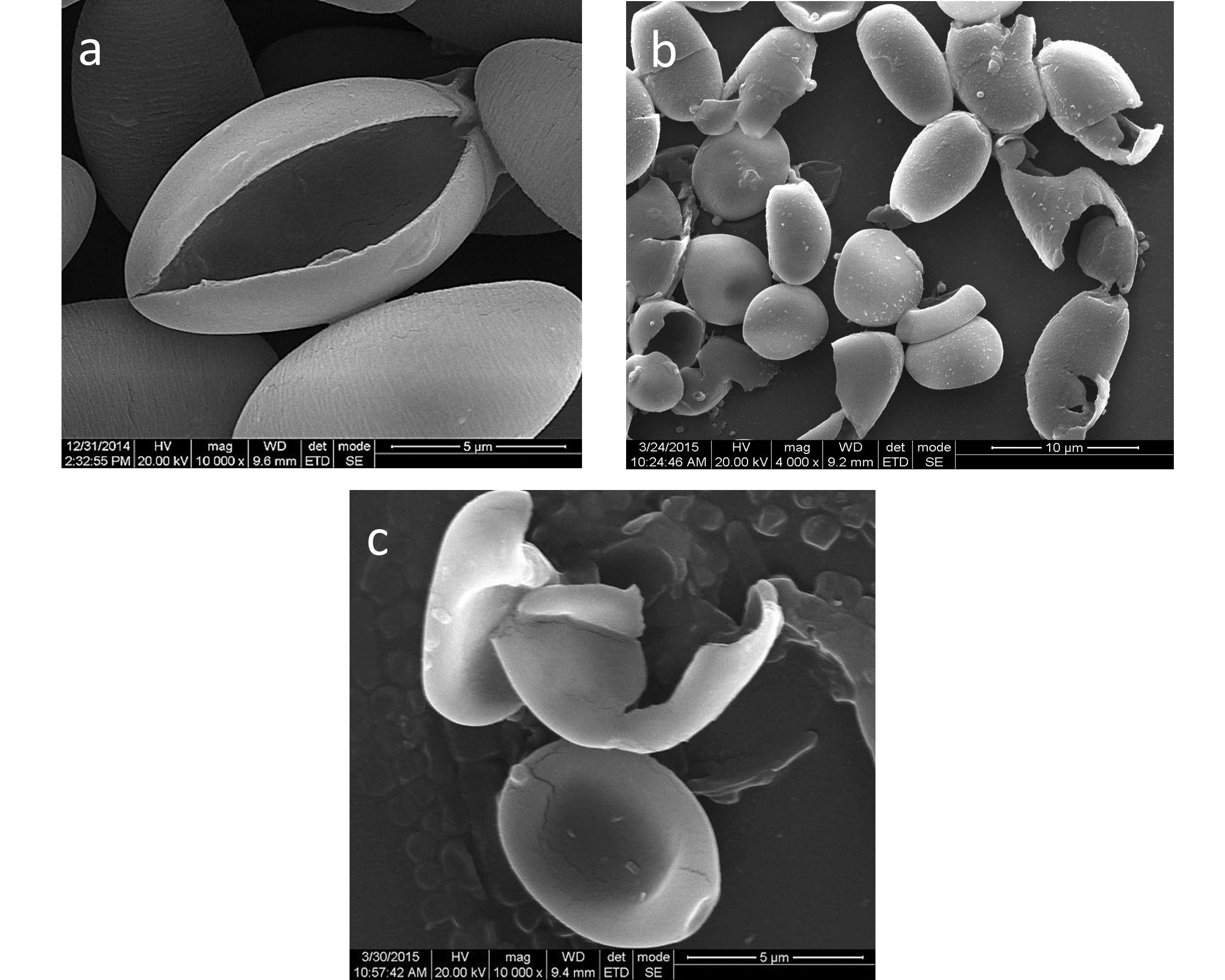

Supplement: S4 Fig — a -Daldinia eschscholzii, b—Coprinellus aureogranulatus and c -Psathyrella gracilis. (TIF) [file pone.0169333.s004.tif]
