# Supplementary material for: Terrestrial Macrofungal Diversity from the Tropical Dry Evergreen Biome of Southern India and Its Potential Role in Aerobiology
Source: PLoS One. 2017 Jan 10;12(1):e0169333. doi: 10.1371/journal.pone.0169333 (PMC5224982; doi:10.1371/journal.pone.0169333)
Supplement: S1 Text — Contains tables (A to F), terminal velocity calculation, fungal spore fall speed calculation and an overview of the macrofungi species observed. (DOCX) [file pone.0169333.s005.docx]

**Supplementary material for**

**Terrestrial macrofungal diversity from the tropical dry evergreen biome of southern India and its potential role in aerobiology**

Hema Priyamvada^1,*^, M. Akila^1^, Raj Kamal Singh^1^, R. Ravikrishna^2^, R. S.Verma^3^, Ligy Philip ^1^, R. R. Marathe^4^, L. K. Sahu^5^, K. P.Sudheer^1^, and S. S. Gunthe^1,*^

^1^ EWRE Division, Department of Civil Engineering, Indian Institute of Technology Madras, Chennai – 36, India

^2^ Department of Chemical Engineering, Indian Institute of Technology Madras, Chennai – 600036, India

^3^ Department of Biotechnology, Indian Institute of Technology Madras, Chennai – 600036, India

^4^ Department of Management Studies, Indian Institute of Technology Madras, Chennai – 600036, India

^5^ Physical Research Laboratory, Navarangpura, Ahmedabad – 380009, India

*To whom correspondence should be sent:

Hema P. ([hema8689@gmail.com](mailto:hema8689@gmail.com))

Sachin S Gunthe ([s.gunthe@iitm.ac.in](mailto:s.gunthe@iitm.ac.in))

**Table A: Detailed description of macrofungi investigated from tropical dry evergreen biome in southern tropical Indian region:** Families identified, species commonly observed, the other part of the world where existence of respective species is reported, pathogenic ability of given species, dwelling nature, economical application, and presence in ambient air as primary biological aerosol particle. The results have been obtained using advanced genetic analysis for 165 samples collected over study region. For the detailed morphological structure of some of the species kindly refer to main text.

| **S. No** | **Family** | **Species** | **Regions of existence** | **Saprophytic/ Pathogenic/ Symbiotic** | **Ground -dwelling (GD)/ Tree - dwelling (TD)** | **Edibility/Toxicity/ Allergenicity/ Medicinal/ Rarity** | **Aerosolization** | **GenBank accession number** |
| --- | --- | --- | --- | --- | --- | --- | --- | --- |
| 1 | *Agaricaceae* | *Agaricus bohusii* | Serbia and southern Europe | Saprophytic | GD | Edible | No record | KU366692 |
| 2 | *Agaricaceae* | *Agaricus dulcidulus* | Italy, Romania and Queensland. | No information is available | GD | Considered to be edible | No record | KU366693 |
| 3 | *Agaricaceae* | *Agaricus fuscofibrillosus* | British Isles, Northern Europe, Southern Europe and Southern America. | Saprophytic | GD and TD | No information is available | No record | KU366694 |
| 4 | *Agaricaceae* | *Agaricus heterocystis* | Chennai | No information is available | GD | No information is available | No record | KU366695 |
| 5 | *Agaricaceae* | *Agaricus hondensis* | Pacific Coast of North America, British Columbia in Canada and California | Saprophytic | GD | Highly poisonous | No record | KU847884 |
| 6 | *Agaricaceae* | *Agaricus moelleri* | North America, Britain, and Europe | Saprophytic | GD | Poisonous | No record | KU847885 |
| 7 | *Agaricaceae* | *Agaricus purpurellus* | Europe, North America and Asia | No information is available | GD | Poisonous | No record | KU847886 |
| 8 | *Lyophyllaceae* | *Calocybe indica* | India and Britian | No information is available | GD | Edible | No record | KU499921 |
| 9 | *Agaricaceae* | *Chlorophyllum globosum* | No information available | No information is available | GD | Poisonous | No record | KU499923 |
| 10 | *Agaricaceae* | *Chloropyhllum molybdites* | North America and California | Saprophytic | GD | Highly poisonous | No record | KU499924 |
| 11 | *Agaricaceae* | *Chlorophyllum nothorachodes* | Australia | No information is available | GD | No information is available | No record | KU499925 |
| 12 | *Entolomataceae* | *Clitopilus giovanellae* | Italy ,Spain and Bulgaria | Saprophytic | GD | Rare | No record | KU712510 |
| 13 | *Entolomataceae* | *Clitopilus scyphoides* | United Kingdom, Belgium, Norway, Sweden, Denmark, Germany and in the United States of America. | Saprophytic | GD | Very rare | No record | KU712511 |
| 14 | *Bolbitiaceae* | *Conocybe dumetorum* | No information available | Saprophytic | GD | Presumed to be poisonous | No record | KU712512 |
| 15 | *Bolbitiaceae* | *Conocybe mandschurica* | Russia | Saprophytic | GD | Antibacterial and enzyme-inhibiting compounds. | Reported | KU712513 |
| 16 | *Psathyrellaceae* | *Coprinellus aureogranulatus* | Papua New Guinea | Saprophytic | GD | No information is available | Reported | KU712514 |
| 17 | *Psathyrellaceae* | *Coprinellus radians* | North America | Saprophytic | GD | No information is available | No record | KU712515 |
| 18 | *Psathyrellaceae* | *Coprinopsis sclerotiorum* | Hawaii | Saprophytic | GD | No information is available | No record | KU712516 |
| 19 | *Psathyrellaceae* | *Coprinopsis spelaiophila* | Antarctica, Austria, Denmark, Germany, Ireland, Spain, Sweden and United Kingdom | Saprophytic | GD | No information is available | No record | KU712517 |
| 20 | *Psathyrellaceae* | *Coprinus echinosporus* | Belgium and other places of Europe | Saprophytic | GD | Allergic genus | No record | KU712518 |
| 21 | *Agaricaceae* | *Cystolepiota bucknallii* | France, Belgium, Switzerland, Austria, Germany, Denmark, Norway, Sweden and Russia | Saprophytic | GD | Very rare and poisonous | No record | KU847887 |
| 22 | *Cortinariaceae* | *Gymnopilus lepidotus* | from Florida, and other parts of USA, Argentina and Mexico. | Saprophytic | GD | Allergic | No record | KU712539 |
| 23 | *Cortinariaceae* | *Gymnopilus purpureosquamulosus* | Italy, Nigeria, Panama, and Switzerland | Saprophytic | GD | No information is available | No record | KU712540 |
| 24 | *Omphalotaceae* | *Gymnopus gibbosus* | Australia | Saprophytic | GD | No information is available | Reported | KU712541 |
| 25 | *Omphalotaceae* | *Gymnopus luxurians* | Massachusetts, Alabama and Illinois | Saprophytic | GD | No information is available | Reported | KU712542 |
| 26 | *Omphalotaceae* | *Gymnopus menehune* | Hawaii, Indonesia and Pakistan | Saprophytic | GD | No information is available | Reported | KU712543 |
| 27 | *Agaricaceae* | *Hymenagaricus epipastus* | Kerala and Sri Lanka | Saprophytic and Plant pathogen | GD | No information is available | No record | KU712544 |
| 28 | *Agaricaceae* | *Hymenagaricus taiwanensis* | Taiwan | No information is available | GD | No information is available | No record | KU847888 |
| 29 | *Agaricaceae* | *Hymenogloea papyracea* | Tropical America, Colombia and Venezuela | No information is available | GD | No information is available | No record | KU847889 |
| 30 | *Agaricaceae* | *Lepiota atrodisca* | California, Pacific coast of States, Santa Cruz and Himachal Pradesh of India. | Saprophytic | GD | Presumed to be poisonous | No record | KU847890 |
| 31 | *Agaricaceae* | *Lepiota castanea* | Europe | Saprophytic | GD | Highly poisonous | No record | KU847891 |
| 32 | *Agaricaceae* | *Lepiota echinacea* | Britain, Ireland and Italy | Saprophytic | GD | Poisonous | No record | KU712547 |
| 33 | *Agaricaceae* | *Lepiota ochraceofulva* | United Kingdom, Sweden, and Germany | Saprophytic | GD | Highly poisonous | No record | KU712548 |
| 34 | *Agaricaceae* | *Lepiota phaeosticta* | Florida | Saprophytic | GD | Highly poisonous | No record | KU712549 |
| 35 | *Tricholomataceae* | *Lepista densifolia* | Norway and Central European mountains | Saprophytic | GD | No information is available | No record | KU847907 |
| 36 | *Tricholomataceae* | *Lepista flaccida* | Europe and North America | Saprophytic | GD | No information is available | No record | KU847908 |
| 37 | *Tricholomataceae* | *Lepista nuda* | North America | Saprophytic | GD | Allergies in sensitized individual when consumed | No record | KU712550 |
| 38 | *Agaricaceae* | *Leucoagaricus atrodisca* | California | Saprophytic | GD | Highly poisonous | No record | KU847892 |
| 39 | *Agaricaceae* | *Leucoagaricus bresadolae* | Taiwan and Japan | Saprophytic | GD | Presumed to be poisonous | No record | KU712551 |
| 40 | *Agaricaceae* | *Leucoagaricus cepaestipes* | North America, Europe and is common in the Eastern parts of the world | Saprophytic | GD | Presumed to be poisonous | No record | KU712552 |
| 41 | *Agaricaceae* | *Leucoagaricus meleagris* | North America, Britian and Europe | Saprophytic | GD | Presumed to be poisonous | No record | KU712553 |
| 42 | *Agaricaceae* | *Leucoagaricus rubrotinctus* | North America | Saprophytic | GD | No information is available | No record | KU712554 |
| 43 | *Agaricaceae* | *Leucoagaricus tangerinus* | China | Saprophytic | GD | No information is available | No record | KU712555 |
| 44 | *Agaricaceae* | *Leucoagaricus vassiljevae* | Russia and Gujarat, India | Saprophytic | GD | Poisonous | No record | KU712556 |
| 45 | *Marasmiaceae* | *Marasmiellus palmivorus* | Malaysia, Indonesia and Hawaii | Saprophytic and Plant pathogen | GD and TD | No information is available | No record | KU712558 |
| 46 | *Marasmiaceae* | *Marasmius albimyceliosus* | Thailand and Singapore | Saprophytic | GD | Inedible | No record | KU712559 |
| 47 | *Marasmiaceae* | *Marasmius haematocephalus* | Africa, Northern Thailand, Malaysia, China, Kerala (India), Ceylon, Surinam, Brazil, Guiana, Cuba and U.S | Saprophytic | GD | Inedible | No record | KU752322 |
| 48 | *Marasmiaceae* | *Marasmius nigrodiscus* | North America | Saprophytic | GD | Inedible | No record | KU752323 |
| 49 | *Marasmiaceae* | *Marasmius nummularius* | Malaysia and North American mountains | Saprophytic | GD | Inedible | No record | KU752324 |
| 50 | *Marasmiaceae* | *Marasmius occultatiformis* | Russia, Siberia and Korea | Saprophytic | GD | Inedible | No record | KU752325 |
| 51 | *Marasmiaceae* | *Marasmius ochroleucus* | Russia, New Caledonia and Northern Thailand | Saprophytic | GD | No information is available | No record | KU752326 |
| 52 | *Marasmiaceae* | *Marasmius oreades* | North America and Europe | Saprophytic | GD | Edible | No record | KU847896 |
| 53 | *Marasmiaceae* | *Marasmius trichotus* | Northern Thailand, Papua New Guinea and Singapore | Saprophytic | GD | Inedible | No record | KU847897 |
| 54 | *Agaricaceae* | *Micropsalliota alba* | Thailand and Kerala | No information is available | GD | No information is available | No record | KU752328 |
| 55 | *Agaricaceae* | *Micropsalliota globocystis* | Singapore, Thailand and Kerala | No information is available | GD | No information is available | No record | KU752329 |
| 56 | *Agaricaceae* | *Micropsalliota lateritia* | Thailand | No information is available | GD | No information is available | No record | KU752330 |
| 57 | *Marasmiaceae* | *Omphalotus japonicus* | Japan and Eastern Asia | Saprophytic | TD | Poisonous | No record | KU752331 |
| 58 | *Marasmiaceae* | *Omphalotus olivascens* | California and Mexico | Saprophytic | TD | Poisonous | No record | KU847898 |
| 59 | *Marasmiaceae* | *Omphalotus subilludens* | Europe, North & Central America | Saprophytic | TD | Poisonous | No record | KU847899 |
| 60 | *Tricholomataceae* | *Paralepista gilva* | Morocco, France, Spain, and Italy | Saprophytic | GD | Poisonous | No record | KU752332 |
| 61 | *Psathyrellaceae* | *Parasola auricoma* | Europe, North America and Hawaii | No information is available | GD | No information is available | No record | KU847905 |
| 62 | *Strophariaceae* | *Pholiota spumosa* | Canada | Saprophytic and Plant pathogen | GD | No information is available | No record | KU847906 |
| 63 | *Pleurotaceae* | *Pleurotus djamor* | Bihar, India | Symbiont | TD | Edible | Reported | KU752336 |
| 64 | *Pluteaceae* | *Pluteus petasatus* | North America | Saprophytic | GD | Edible | No record | KU752337 |
| 65 | *Pluteaceae* | *Pluteus romelii* | Europe and North America | Saprophytic | GD | Neurotoxic to humans | No record | KU752338 |
| 66 | *Pluteaceae* | *Pluteus thomsonii* | North America, and documented in California | Saprophytic | GD | No information is available | No record | KU847900 |
| 67 | *Psathyrellaceae* | *Psathyrella candolleana* | Europe, North America and Iraq | Saprophytic | GD | Poisonous | Reported | KU752340 |
| 68 | *Psathyrellaceae* | *Psathyrella gracilis* | New Zealand, North America and Europe | Saprophytic | GD | Presumed to be poisonous | Reported | KU752341 |
| 69 | *Schizophyllaceae* | *Schizophyllum commune* | Mizoram, North America and throughout the world | Saprophytic and Plant pathogen | TD | Respiratory aeroallergen | No record | KU752343 |
| 70 | *Clavariaceae* | *Scytinopogon angulisporus* | Mexico and West Bengal | Symbiont | GD | No information is available | No record | KU847894 |
| 71 | *Lyophyllaceae* | *Termitomyces heimii* | Tropical and subtropical areas of Africa and Asia. Has been widely reported from India | Symbiont | GD | No information is available | No record | KU847895 |
| 72 | *Lyophyllaceae* | *Termitomyces microcarpus* | Africa and India - Goa, Karnataka and in the south-western region of the Western Ghats | Saprophytic | GD | Edible | No record | KU752344 |
| 73 | *Tricholomataceae* | *Tricholoma mongolicum* | North China and Mongolia | Symbiont | TD | Medicinal and edible | Reported | KU752348 |
| 74 | *Pluteaceae* | *Volvariella dunensis* | Mediterranean coast of Spain | Saprophytic | GD | No information is available | No record | KU847901 |
| 75 | *Pluteaceae* | *Volvariella hypopitys* | No available information yet | Saprophytic | GD | No information is available | No record | KU847902 |
| 76 | *Pluteaceae* | *Volvariella taylorii* | Pacific North West | Saprophytic | GD | No information is available | No record | KU752351 |
| 77 | *Phanerochaetachae* | *Ceriporia lacerata* | Europe, North and South America, Australia and U.K | Saprophytic | TD | Respiratory aeroallergen | No record | KU499922 |
| 78 | *Polyporaceae* | *Coriolopsis byrsina* | Tropical east Africa | Saprophytic | GD | No information is available | Reported | KU712519 |
| 79 | *Polyporaceae* | *Coriolopsis caperata* | North America | Saprophytic and Plant pathogen | TD | No information is available | Reported | KU712520 |
| 80 | *Polyporaceae* | *Coriolopsis gallica* | North America | Saprophytic and Plant pathogen | TD | No information is available | Reported | KU847903 |
| 81 | *Polyporaceae* | *Dichomitus squalens* | South west of America | Saprophytic and Plant pathogen | TD | Occurs only in marine environment. Rare occurrence in other environment | No record | KU712525 |
| 82 | *Polyporaceae* | *Earliella scabrosa* | Taiwan | Saprophytic and Plant pathogen | TD | No information is available | No record | KU712526 |
| 83 | *Polyporaceae* | *Favolus emericii / Polyporus emericii* | Philippines, Ceylon, Australia, New Zealand, Cuba, New Guinea and Brazil | Saprophytic and Plant pathogen | TD | No information is available | No record | KU712527 |
| 84 | *Meruliaecea* | *Flavodon flavus* | No information available | Saprophytic and Plant pathogen | TD | No information is available | No record | KU712528 |
| 85 | *Polyporaceae* | *Fomes fomentarius* | Europe, Asia, Africa and North America | Saprophytic and Plant pathogen | TD | Medicinal | No record | KU712529 |
| 86 | *Ganodermataceae* | *Ganoderma applanatum* | England and United states | Saprophytic and Plant pathogen | TD | No information is available | Reported | KU712532 |
| 87 | *Ganodermataceae* | *Ganoderma carnosum* | Europe | Saprophytic and Plant pathogen | TD | No information is available | Reported | KU712533 |
| 88 | *Ganodermataceae* | *Ganoderma lucidum* | Eastern countries and many Asian countries | Saprophytic and Plant pathogen | TD | Medicinal, but the spores cause allergies in sensitized individuals | Reported | KU712534 |
| 89 | *Ganodermataceae* | *Ganoderma multipileum* | Tropical Asian countries | Saprophytic and Plant pathogen | TD | Medicinal | Reported | KU712535 |
| 90 | *Ganodermataceae* | *Ganoderma neojaponicum* | Mainland China, Japan and Taiwan | Saprophytic and Plant pathogen | TD | Medicinal | Reported | KU712536 |
| 91 | *Ganodermataceae* | *Ganoderma tsugae* | China and New Mexico | Saprophytic and Plant pathogen | TD | Medicinal | Reported | KU712531 |
| 92 | *Polyporaceae* | *Lenzites elegans* | Southeastern United States and all other tropical regions | Pathogenic | TD | No information is available | No record | KU712546 |
| 93 | *Polyporaceae* | *Lopharia cinerascens* | United States, Canada, Cuba, Jamaica, Mexico, Venezuela and South Africa | Noinformation is available | TD | No information is available | No record | KU712557 |
| 94 | *Polyporaceae* | *Microporus ochrotinctus* | Pacific Islands | Noinformation is available | TD | No information is available | No record | KU752327 |
| 95 | *Polyporaceae* | *Perenniporia fraxinea* | Northern and Central Europe | Saprophytic and a perennial Plant pathogen | TD | No information is available | No record | KU847904 |
| 96 | *Polyporaceae* | *Perenniporia ochroleuca* | Madeira island of Portugal and China | Saprophytic and Plant pathogen | TD | No information is available | No record | KU752333 |
| 97 | *Polyporaceae* | *Polyporus grammocephalus* | West Bengal, India | Saprophytic | TD | No information is available | No record | KU752339 |
| 98 | *Polyporaceae* | *Trametes elegans* | Tropical regions of world and Nigerian forests | Saprophytic and Plant pathogen | TD | No information is available | Reported | KU752345 |
| 99 | *Polyporaceae* | *Trametes robiniophila* | No information available | Saprophytic and Plant pathogen | TD | Medicinal | No record | KU752346 |
| 100 | *Polyporaceae* | *Trametes suaveolens* | United Kingdom and Netherlands | Saprophytic and Plant pathogen | TD | No information is available | No record | KU752347 |
| 101 | *Polyporaceae* | *Truncospora macrospora* | South west China | Symbiont | TD | No information is available | No record | KU752349 |
| 102 | *Polyporaceae* | *Truncospora ochroleuca* | Africa, London, Ceylon, Japan, Australia, Tasmania and New Zealand | Saprophytic and Plant pathogen | TD | No information is available | No record | KU752350 |
| 103 | *Hymenochaetaceae* | *Fulvifomes fastuosus* | Sri Lanka,West Pakistan and Tokyo. | Symbiont | TD | No information is available | No record | KU712530 |
| 104 | *Hymenochaetaceae* | *Phellinus repandus* | Western United states | Saprophytic and Plant pathogen | TD | No information is available | No record | KU752335 |
| 105 | *Hymenochaetaceae* | *Pyrrhoderma scaurum* | China (Zhejiang), Japan (Honshu, Hokkaido), and Far East Russia | Symbiont | TD | No information is available | Reported | KU752342 |
| 106 | *Geastraceae* | *Geastrum pectinatum* | London and Ireland | Saprophytic | GD | Inedible | Possible | KU712537 |
| 107 | *Geastraceae* | *Geastrum striatum* | Leicestershire and Rutland | Saprophytic | GD | Inedible | Possible | KU712538 |
| 108 | *Auriculariaceae* | *Auricularia fuscosuccinea* | Western Ghats of Kerala | Symbiont | TD | No information is available | No record | KU847893 |
| 109 | *Auriculariaceae* | *Auricularia polytricha* | Western Ghats of Kerala | Symbiont | TD | Medicinal and edible | No record | KU499920 |
| 110 | *Nectriaceae* | *Cosmospora viliuscula* | No information available | Saprophytic and Plant pathogen | TD | No information is available | No record | KU712522 |
| 111 | *Xylariaceae* | *Daldinia eschscholzii* | Australia, Russia and Gujarat | Saprophytic and Plant pathogen | TD | No information is available | Reported | KU712524 |
| 112 | *Xylariaceae* | *Hypoxylon rickii* | No information available | Saprophytic and Plant pathogen | TD | No information is available | No record | KU712545 |
| 113 | *Xylariaceae* | *Xylaria cirrata* | Taiwan | Saprophytic | GD | No information is available | No record | KU752352 |

**Table B: Sampling details of the study region (Chennai, IITM) and other regions of study.** Details regarding their sampling method, sampling season, vegetation, sampling area and the number of samples collected are provided in the table.

| **Location** | **Sampling type and season** | **Forest type** | **Location details and sampling area** | **Macrofungi sampled** | **Reference** |
| --- | --- | --- | --- | --- | --- |
| **Chennai** | Opportunistic random sampling during north - east monsoon | Tropical dry evergreen biome | Study area is 687 acres in total, of which 80% of the area was, sampled which stands to be 550 acres. It is to be noted that, even though the study region is a forest region there is significant human occupation and activity | 165 samples | Current study |
| **Karnataka** | Repeated random sampling during the south - west monsoon | Semi-evergreen and moist deciduous forests of Shimoga | Selected areas of forests were sampled by diving the forest area into 100 transects with area as 50 m* 20m each. Thus the effective studied area was 25 acres | 778 samples | (1) |
| **West Bengal** | Random sampling during south - west monsoon | Tropical dry deciduous forests of lateritic regions of West Bengal | Forest under study was divided into 90 quadrats of 20m*20m area each. Thus the effective studied area is 9 acres | 120 samples | (2) |
| **Kerala** | Opportunistic random sampling during south - west, north - east and post monsoon seasons | Forest is a mixture of tropical evergreen, semi - evergreen, moist deciduous, sub - tropical broad leaved, montane wet tempered, tropical dry deciduous, grasslands and swamp forest type | Four regions of the forest were selected each with an area of 100m*100m each. Thus the effective study area is 10 acres | 616 samples | (3,4) |
| **Maharashtra** | Random sampling during south - west monsoon | Western Ghats evergreen forest regions of Mahabaleshawar and Mulshi | Random sample collection covering 137 km2, which is 33890 acres being covered for the study | 178 samples | (5,6) |

**Table C: Description about the spread of various families of macrofungi reported over four other parts of India (Karnataka, West Bengal, Maharashtra, and Kerala).** The spread in distribution amongst the families for these locations was calculated using a Chi-square test. Please refer to the main text for more details about the methodology. Please note that the numbers in the upper part of the table indicated the absolute observed frequency whereas lower part indicates the expected frequency for respective families.

| **Sampled observation - Observed Frequency** | | | | | |
| --- | --- | --- | --- | --- | --- |
| Common families (obs) | Chennai | Karnataka | West Bengal | Maharashtra | Kerala |
| *Agaricaceae* | 28 | 41 | 5 | 27 | 129 |
| *Marasmiaceae* | 13 | 19 | 6 | 13 | 60 |
| *Pluteaceae* | 6 | 6 | 6 | 8 | 34 |
| *Polyporaceae* | 18 | 73 | 8 | 19 | 15 |
| *Others* | 48 | 119 | 12 | 26 | 155 |
| Sum | 113 | 258 | 37 | 93 | 393 |
| **Sampled observation - Expected Frequency** | | | | | |
| Common families (obs) | Chennai | Karnataka | West Bengal | Maharashtra | Kerala |
| *Agaricaceae* | 29.1 | 67.4 | 9.7 | 24.3 | 102.7 |
| *Marasmiaceae* | 14 | 32.5 | 4.7 | 11.7 | 49.6 |
| *Pluteaceae* | 7.6 | 17.6 | 2.5 | 6.3 | 26.8 |
| *Polyporaceae* | 16.8 | 39.0 | 5.6 | 14.1 | 59.4 |
| *Others* | 45.5 | 101.4 | 14.5 | 36.6 | 154.5 |
| Sum | 99.0 | 258.0 | 37.0 | 93.0 | 393.0 |
| **Simulated/resampled -Observed Frequency** | | | | | |
| Common families (res) | Chennai | Karnataka | West Bengal | Maharashtra | Kerala |
| *Agaricaceae* | 28 | 41 | 5 | 27 | 129 |
| *Marasmiaceae* | 10 | 19 | 6 | 13 | 60 |
| *Pluteaceae* | 6 | 6 | 6 | 8 | 34 |
| *Polyporaceae* | 18 | 73 | 8 | 19 | 15 |
| *Others* | 65 | 119 | 12 | 26 | 155 |
| Sum | 127 | 258 | 37 | 93 | 393 |
| **Simulated/resampled - Expected Frequency** | | | | | |
| Common families (res) | Chennai | Karnataka | West Bengal | Maharashtra | Kerala |
| *Agaricaceae* | 32.2 | 65.4 | 9.4 | 23.6 | 99.5 |
| *Marasmiaceae* | 15.1 | 30.7 | 4.4 | 11.1 | 46.7 |
| *Pluteaceae* | 8.4 | 17.0 | 2.4 | 6.1 | 26.0 |
| *Polyporaceae* | 18.6 | 37.8 | 5.4 | 13.6 | 57.6 |
| *Others* | 52.7 | 107.1 | 15.4 | 38.6 | 163.2 |
| Sum | 127.0 | 258.0 | 37.0 | 93.0 | 393.0 |

**Table D: Description related to the morphology and ornamentation of spores of some the selective species obtained using Scanning Electron Microscopy (SEM) imaging**. Family, species, average size, approximate shape, typical and specific features, and nature of spore surface.

| **S.No** | **Family** | **Species** | **Size** | **Shape** | **Attachment scar** | **Nature of wall** | **Fig no** |
| --- | --- | --- | --- | --- | --- | --- | --- |
| 1 | *Agaricaceae* | *Agaricus hondensis* | 4.5-6 x 3-4 µm | ellipsoidal | Conspicuous apiculus | Smooth | 3.a |
| 2 | *Agaricaceae* | *Agaricus moelleri* | 4–6 x 3–4 µm | globose to ellipsoidal | Conspicuous apiculus | Smooth | 3.b |
| 3 | *Agaricaceae* | *Chlorophyllum nothorachodes* | 6-8 x 5-7 µm | ellipsoidal to oblong | Conspicuous apiculus | Smooth | 3.c |
| 4 | *Bolbitiaceae* | *Conocybe mandschurica* | 4–8 x 4.5–6 µm | ellipsoidal | Conspicuous apiculus | Smooth | 3.d |
| 5 | *Psathyrellaceae* | *Coprinellus aureogranulatus* | 6.2-7.8 x 4.1-5.1 µm | sub-cylindrical to ellipsoidal | Conspicuous apiculus | Smooth | 3.e |
| 6 | *Psathyrellaceae* | *Coprinellus radians* | 6-10 x 4.5-6 µm | cylindrical to ellipsoidal | Conspicuous apiculus | Smooth | 3.f |
| 7 | *Cortinariaceae* | *Gymnopilus purpureosquamulosus* | 6-8 x 4-4.5µm | ellipsoidal to oblong | Conspicuous apiculus | Warty | 3.g |
| 8 | *Agaricaceae* | *Hymenagaricus taiwanensis* | 4-9x 4-5 µm | ellipsoidal | Conspicuous apiculus | coarsely verrucose | 3.h |
| 9 | *Agaricaceae* | *Leucoagaricus atrodisca* | 4-6 x 3.5-5 µm | ellipsoidal | Conspicuous apiculus | Smooth | 3.i |
| 10 | *Agaricaceae* | *Micropsalliota globocystis* | 5-10 x 2-4 µm | ellipsoidal | Conspicuous apiculus | Smooth and hyaline | 3.j |
| 11 | *Strophariaceae* | *Pholiota spumosa* | 6.0-9.5 x 4.0-5.5 µm | ellipsoidal to oblong-ellipsoidal | Conspicuous apiculus | coarsely verrucose | 3.k |
| 12 (a) | *Psathyrellaceae* | *Psathyrella candolleana (young)* | 6.5-9.5 x 4-5 µm | ellipsoidal | Conspicuous apiculus | Smooth | 3.l |
| 12 (b) | *Psathyrellaceae* | *Psathyrella candolleana (mature)* | 6.5-9.5 x 4-5 µm | ellipsoidal | Conspicuous apiculus | Smooth, slightly wrinkled and shrunken | 3.m |
| 13 | *Psathyrellaceae* | *Psathyrella gracilis* | 5-5.5 x 4-5 µm | ellipsoidal | Conspicuous apiculus | Smooth | 3.n |
| 14 | *Pluteaceae* | *Volvariella taylorii* | 5.6-8.7 x 4.1-6 μm | ellipsoidal | Conspicuous apiculus | Smooth | 3.o |
| 15 | *Phanerochaetachae* | *Ceriporia lacerata* | 5-5.5 × 4-5 μm | ellipsoidal to oblong | Conspicuous apiculus | Smooth and hyaline | 3.p |
| 16 | *Ganodermataceae* | *Ganoderma lucidum* | 8-6 × 5-8 μm | ellipsoidal | Conspicuous apiculus | Slightly wrinkled | 3.q |
| 17 | *Hymenochaetaceae* | *Phellinus repandus* | 3-4 x 3-5 µm | ovoid to sub-globose | Conspicuous apiculus | Smooth | 3.r |
| 18 | *Geastraceae* | *Geastrum pectinatum* | 3-4 x 4-5 µm | globose | Inconspicuous apiculus | Warty | 3.s |
| 19 | *Geastraceae* | *Geastrum striatum* | 3-4 x 4-5μm | spherical | Conspicuous apiculus | coarsely verrucose | 3.t |
| 20 | *Nectriaceae* | *Cosmospora viliuscula* | 10-10.5 × 5–5.5 μm | Ellipsoidal | Inconspicuous apiculus | Smooth | 3.u |
| 21 | *Xylariaceae* | *Daldinia eschscholzii* | 13-15 x 6-7 µm | ellipsoid-inequilateral | Inconspicuous apiculus | mildly striated | 3.v |
| 22 | *Xylariaceae* | *Xylaria cirrata* | 4–5 × 2–2.5 μm | fusoid to ellipsoid | Conspicuous apiculus on the less convex side | Wrinkled | 3.w |

**Table E:** Terminal velocity and fall speed of spores of different size

| Fungal spore diameter (µm) | Settling velocity (m/S) | Time to fall 1 meter (min) | Time to fall 10 meter (min) | Time to fall 15 meter (min) |
| --- | --- | --- | --- | --- |
| 3 | 0.00109 | 15.294 | 153.213 | 229.406 |
| 5 | 0.00303 | 5.506 | 55.157 | 82.586 |
| 7 | 0.00593 | 2.809 | 28.141 | 42.136 |
| 9 | 0.00981 | 1.699 | 17.024 | 25.490 |
| 11 | 0.01465 | 1.138 | 11.396 | 17.063 |

**Table F: Summary of the meteorological conditions that existed in the study region during the sampling period of October 2014 to February 2015.** Temperature (˚C), Humidity (%) and cumulative rainfall as precipitation (mm) has been represented. Study region receives rainfall from the North-east monsoon during October – December, which is also the winter season.

| Season | Month | Temperature (˚C) | Humidity (%) | Precipitation (mm) |
| --- | --- | --- | --- | --- |
| Winter & Rainfall | October | High - 32 | High - 95.5 | Sum - 103.12 |
|  |  | Avg - 28.3 | Avg - 81 |  |
|  |  | Low - 24.4 | Low - 55.1 |  |
| Winter & Rainfall | November | High - 30 | High - 95.2 | Sum - 29.22 |
|  |  | Avg - 25.6 | Avg - 80 |  |
|  |  | Low - 22.6 | Low - 54 |  |
| Winter & Rainfall | December | High - 29 | High - 96 | Sum - 123.45 |
|  |  | Avg - 25.6 | Avg - 80.1 |  |
|  |  | Low - 22.1 | Low - 55.6 |  |
| Winter | January | High - 29.2 | High - 96.1 | Sum - 4.07 |
|  |  | Avg - 25 | Avg - 76 |  |
|  |  | Low - 20.5 | Low - 48.1 |  |
| Summer | February | High - 31 | High - 93.4 | Sum - 0 |
|  |  | Avg - 26 | Avg - 71 |  |
|  |  | Low - 21 | Low - 21 |  |

**Table References:**

1. Swapna S SA and KM. Diversity of Macrofungi in Semi-Evergreen and Moist Deciduous Forest of. J Mycol Pl Pathol. 2008;38(1):21–6.

2. Pradhan P, Dutta AK, Roy A, Basu SK, Acharya K. Macrofungal diversity and habitat specificity: a case study. Biodiversity [Internet]. 2013;14(3):147–61. Available from: http://www.tandfonline.com/doi/abs/10.1080/14888386.2013.805660

3. Farook V, Khan S, Manimohan P. A checklist of agarics (gilled mushrooms) of Kerala State, India. Mycosphere [Internet]. 2012;4(1):97–131. Available from: http://www.mycosphere.org/pdfs/MC4_1_No6.pdf

4. Mohanan C. Macrofungal diversity in the Western Ghats , Kerala , India : members of Russulaceae. J Threat Taxa. 2014;6(April):5636–48.

5. Senthilarasu G. Diversity of agarics (gilled mushrooms) of Maharashtra, India. Curr Res Environ Appl Mycol. 2014;4(1):58–78.

6. Kolet M. Mushrooms And Macrofungi From Jnanadweepa , College Campus in Thane ,. In: National Conference on Biodiversity : Status and Challenges in Conservation - “FAVEO” 2013. 2013. p. 184–8.

**Calculating the terminal velocity and the fall speed of fungal spores:**

Since majority of the fungal spores are dispersed through air, the behavior of spores when suspended in air is of special interest to the scientists working in the fungal aerobiology. A significant characteristic is the rate of fall in still air and it is called as the terminal velocity. For a spherical spore, fall in still air is governed by Stoke’s law which is expressed in the equation as (1),

$$V=\frac{2}{9} . \frac{\sigma- \rho}{\mu} . gr^{2}$$

Where, V is steady terminal velocity in cm/s,

$\sigma$ is density of spore,

$\rho$ is density of air,

g is acceleration due to gravity ( 981cm/s),

µ is viscosity of air (1.8 × 10^-4^ g/cm/s at 18˚C), and

r is radius of the spore.

Since the density of the spore is approximately 1.0 and that of the air is so small that it is ignored and the equation is simplified as,

$$V=\frac{2gr^{2}}{9\mu}$$

Thus the rate of the fall of a fungal spore is proportional to the square of its radius. It has been proved by scientists that there is a reasonable agreement between theory and observed values (1). From the terminal velocity the fall speed of the fungal spores were calculated for three different distances – 1m, 10m and 15m. The height of 10m is important and justified because it is at the top of the Prandtl layer in which the fluxes between the ground and the atmosphere are constant with the height (2). Thus the concentration of spores at this level would be in a steady state between the spore emission and dry deposition (2). Kindly refer Table S5 for spore terminal velocity and fall speed calculations.

**Overview of species observed:**

In this section we provide an overview of different properties of the individual species. To maintain the brevity the discussion is focused on appearance, spore morphology, habitat, and distribution of all 113 species. For the simplicity the observed species are categorized as phyla, order, family, and species. Note, however, that the description about images obtained using SEM analysis is restricted to certain species. This is mainly due to the fact that difficulties associated with handling, extraction, preparation, changes in operational conditions, etc. while preparing the samples for SEM analysis.

**Basidiomycota**

*Agaricomycetes*

Agaricales

*Agaricus bohusii*

This is a large macrofungi found growing distinctively under the broad-leaved trees and the circular to flat fruiting body includes a cap of diameter 20-30 cm, with length and diameter of stem being 25 cm and 3 cm, respectively. Change in color from reddish brown to dark brown upon physical handling is a very distinctive characteristic of this fungus (3). The spores of this macrofungi are brown colored and egg shaped with the size ranging from 6 to 7 µm on the longer edges. This gregarious macrofungi is an edible and prized fungus (4) and it has been reported earlier in Serbia and southern Europe (3).

*Agaricus dulcidulus*

This fungus is commonly called as the ‘almond mushroom’ due to its peculiar almond resembling smell with a 2 – 7 cm convex and rounded cap (5). Gills or lamella are dense and were reported to have the ability to change color from pale gray-brownish (young) to dark purple-brown (old) as it grows (5). The dark brownish spores had a size range of 4 – 6 µm with typical shape of non-amyloid and elliptical. The gregarious fungus prefers humid soil with rich decomposed leaf litter as its substrate; it has also been reported to grow near the base of tree trunk. This rosy wood fungus has been reported earlier from Italy, Romania, and Queensland (6).

*Agaricus fuscofibrillosus*

This solitude and gregarious fungus is mostly found in the coastal areas growing on the base of the tree. The cap is ~4-15 cm broad, convex and becomes flat as it grows. This specific fungus turns red and causes reddish to brown stains when bruised and lamella is closely packed, which is often brown to black in color with stipe 4 -15 cm tall and ~1.5-2.5 cm thick. Spore print is of dark brownish color and the spores are of the size 5 – 6 µm, smooth and elliptical in shape. Its presence has been reported earlier from coastal California and is most common in the San Francisco Bay Area. It is also found in the British Isles, Northern Europe, Southern Europe and Southern South America (7–10).

*Agaricus heterocystis*

This ground-dwelling edible fungus has been reported earlier from coastal site of Chennai, India and Australia (11,12) , where it was found growing at the bottom of the eucalyptus trees. The fungus is mostly umbonate with its cap being convex and broad. Stipe is long and thin. This fungus has been utilized for many different studies that include the anti-microbial activity, enzyme production and isolation, anti-oxidant activity, etc. (11,13).

*Agaricus hondensis*

This fungus is also called as the ‘felt-ringed agaricus’ and found mainly in the woodlands and mixed forests. It is generally large in appearance and has got white cap with small pinkish grey fibers. Cap is convex, 7.5-15 cm long and 1-2 cm thick with a bulbous base. The lamella is typically whitish at first and turns pink to brown with age. Stipe is 7-20 cm long and 1-2.5 cm thick and slightly thinner at the cap attachment. The presence of a thick ring on the stalk and a peculiar foul smell typically distinguishes this fungus. Spore print is dark brown elliptically-shaped and is 4 – 6 µm in size with smooth texture and conspicuous apiculus (Fig.4a). This highly poisonous fungus has been reported earlier from the Pacific Coast of North America, British Columbia in Canada, and California (14–18).

*Agaricus moelleri*

This inedible saprophytic woodland fungus is also called as the inky mushroom because of the presence of a strong unpleasant odor, which resembles the Indian ink. When consumed mistakenly, it can lead to severe gastro-intestinal problems. The size of the grey colored cap ranges from 5-10 cm with a ring that is present in the bulbous base. The pink colored lamella is tightly packed and turns to brown color with age. The spores are dark brown in color and the spores are 4 – 6 µm in size, with its shape being globose to ellipsoidal (Fig.4b). It has been reported earlier from the temperate zones of northern hemisphere that includes North America, Britain, and Europe (10,19,20).

*Agaricus purpurellus*

This edible fungus has its convex-rounded cap in the size range of 2 to 7 cm and has distinct inflexed margins, which flattens as it grows with a densely packed lamella. The stipe is 2–5 cm long and 0.4–0.8 cm broad and bulbous. Spore print is dark brown and the spores are 4 – 6 µm in diameter and are elliptical in shape. This diminutive agaric is poisonous in nature and commonly found in Europe, North America, and Asia (21).

*Calocybe indica*

This fungus is also called as the ‘milky mushroom’ and is the first macrofungi to be commercialized in India (22). The entire fruiting body, which includes the cap, gills, and stalk, is fully white in color. The cap is mostly convex, getting flatter with age. Gills are adnexed to free. The stipe is thick and broader towards the base. This fungus is considered as one of the best edible fungus that is capable of growing in high temperature ranging from 30 - 35°C (23). This tropical fungus has been reported earlier from India and Britain (24).

*Chlorophyllum globosum*

Macrofungi belonging to the genus *chlorophyllum* is generally poisonous. They prefer rich soil with leaf litter as their substrate for the growth. The cap is convex and thick fleshed with whitish gills. Stipe is slender with a bulbous base. The fungi belonging to the genus *chlorophyllum* have been widely reported to be present in the subtropical regions (25,26).

*Chloropyhllum molybdites*

This fungus is also called as the ‘false parasol’ or ‘green-spored parasol mushroom’. This is extremely poisonous and can cause severe gastrointestinal symptoms of vomiting and diarrhea when consumed mistakenly. This gregarious fungus is found growing in a circular pattern creating a ‘fairy ring’ and this is one of the distinguishing features of this fungus. Cap is 10 – 30 cm in diameter and convex to conical when young and becomes flat with age. The gills are free from stem and are greyish green in color. The stipe is 5 – 25 cm long and 1.5 - 2.5 cm thick. The spore print is green colored and are 8 – 9 µm in size, smooth, elliptical, dextrinoid with a conspicuous apiculus. It has been reported earlier from the subtropical regions of the world and from the grasslands and woodlands of North-Eastern America and California (27–30).

*Chlorophyllum nothorachodes*

This ground – dwelling gregarious macrofungi is generally found in the rich soil of grasslands and woodlands. Pileus extends up to 28 cm and ends with a wavy margin. Lamella is free, crowded and is brown colored. Stipe is 25 cm long, 2.5 – 4 cm wide and appears to have a bulbous base. The off-white to cream colored fruiting body becomes brownish – orange when bruised or damaged. Spores are ellipsoidal to oblong, thick- walled, and range 6 – 8 µm in size. The SEM images of the spores are shown in Fig.4c. This species has been reported earlier from Australia (31).

*Clitopilus giovanellae*

This gregarious ground – dwelling macrofungi is mostly found in the sandy soils with discontinuous vegetation cover. The convex to plane pileus extends 5 – 15 cm in diameter and has got a very lean flesh. Lamella is thin, distant, decurrent, and white to grey colored. Stipe is 10 – 20 × 1 – 2 cm, cylindrical and has got a sub-bulbous base. The basidiocarp of the fruiting body smells like wheat flour. Spore print is white colored with the basidiospores having the dimensions 5 – 8 µm and the shape is ellipsoidal to ovoid. It has been reported earlier from Italy and Spain. This fungus has been reported as the endangered, vulnerable, rare, and indeterminate species of Bulgaria (32,33).

*Clitopilus scyphoides*

This ground-dwelling macrofungi mostly grows on the soil and on woody debris. Pileus is convex shaped in the beginning and flattens with age. The white colored cap has a frosted appearance and extends up to 2 cm. Lamella is decurrent; white colored in the beginning and then becomes pink with age. This fungus is quite rare and has a very meager distribution. Spore print is pink colored and the spores are ellipsoidal and angular with the size range of 5 – 8 µm. It has been reported earlier from United Kingdom, Belgium, Norway, Sweden, Denmark, Germany and in the United States of America (34–36).

*Conocybe dumetorum*

The detailed information about appearance and distribution of this macrofungi is not available. Majority of the fungi belonging to the genus *conocybe*, however, have been reported to have few features common among them. The fungi of this genus are generally found to grow in fertile rich soil, grasslands, and in decayed woodland and almost all of them have delicate and thin stipe. They get their name *conocybe* because of the presence of a cone-shaped cap. The cap is mostly umbonate and becomes flat with age. Gills are dense and cinnamon colored. Stipe is slender and frail. Many of the species belonging to this genus are poisonous and can cause hallucinations (37–39).

*Conocybe mandschurica*

This ground-dwelling macrofungi has its pileus, which is 5 – 8 cm broad, obtusely conical to conical-convex in shape. Pileus is yellow-brown in color with dark reddish center when moist but the color changes to dark-grey to brown when it is dry. Lamella is distant and narrowly adnate. Stipe is 10 – 15 cm long and 1 cm thick, cylindrical with slightly bulbose base. Spores are 4 – 8 µm in size, ellipsoidal in shape and have got a conspicuous apiculus (Fig.4d). This fungus grows on soil rich in leaf litter and has been reported earlier from Russia (40,41).

*Coprinellus aureogranulatus*

This golden brown woolly fungus has its pileus which is 5 cm wide. The white colored free lamella turns black with age. Stipe is 10 cm long and 0.2 – 0.3 cm thick, silky white in color with a slightly bulbous base. Spores are 6 – 7 µm in size, sub-cylindrical to ellipsoidal with a smooth surface and a thick wall (Fig.4e). This fungus has been found growing in the rich soils especially in the tree root areas and has been reported earlier from Papua New Guinea (42,43).

*Coprinellus radians*

This gregarious fungus is commonly called as the ‘Orange-mat coprinus’ as it is found growing as a dense orange mat of mycelia. The tawny-brown to yellowish-orange pileus is 2 – 3 cm wide, felted in texture with a whitish veil that breaks up into small, white to cream woolly flocks (44). Lamella is free; white colored when young and becomes black as it ages with a liquefying inky texture. Spore print is black colored, 6 - 10 µm in diameter and is cylindrical to ellipsoidal in shape (Fig.4f). One striking feature of this fungus is that it grows indoors, especially on moist wooden surfaces. It has been reported earlier from North America (45) .

*Coprinopsis sclerotiorum*

This ground – dwelling macrofungi has its pileus which is 1.4 cm wide and 2 cm long with dark brown plaques of veil that is 5 cm wide. The brown colored pileus becomes grey and deliquescent with age. This fungus is known to possess a distinct fishy odor. Lamella is closely packed and is black in color. The white colored stipe is 3.5 cm long, 0.5 cm wide, with a sub-bulbous base that tapers upward. Spores are 13 - 16 µm in diameter and are sub-globose to clavate. This rare fungus grows in the woodlands and has been reported earlier from Hawaii (46,47).

*Coprinopsis spelaiophila*

This ground – dwelling deliquescing macrofungi is also called as the ‘ink-cap’ because the depleted gills (after shedding all the spores) auto-digests into a clear liquid that becomes black with age. One of the distinctive features of this fungus is the presence of big tufts of veiled remnants on the cap. Pileus and the stipe is white colored. It has been reported earlier from Antarctica, Austria, Denmark, Germany, Ireland, Spain, Sweden and United Kingdom (48–50).

*Coprinus echinosporus*

This ground - dwelling macrofungi has an ellipsoidal to ovoid pileus that has the dimensions of 1 - 2 x 0 .5-1 cm when still closed, it is 1 - 3 cm when expanded. Pileus is white colored when young, becomes grey with age and the veils present on the top of the fungus are white to grey colored. Lamella is free or crowded and the color changes from white to grey with age. Stipe is 10 cm long and 0.1 - 0.3 cm wide with a slightly clavate base. Spores are 8 - 12 µm in diameter, amygdaliform, warty with a conspicuous apiculus. This fungus found mostly on the tree litter and tree stumps, has been reported earlier from Belgium with a meager distribution in the other parts of Europe (51–53).

*Cystolepiota bucknallii*

This is a very rare and poisonous ground- dwelling macrofungi that is a thin fleshed agaric. This non-edible fungus has a distinctive smell of coal gas and is normally found in the damp soil. The lilac colored cap which is 2–4cm wide changes its shape from hemisphere to convex, with age. Lamella is crowded and is not attached to the stipe, which is 2 - 4.5cm long and 0.3 – 0.5cm wide. A mealy powder covers the fruiting body and the stipe. Spores are elongated, ellipsoidal in shape with the diameter as 7-10 µm. It has been reported from central and northern Europe including France, Belgium, Switzerland, Austria, Germany, Denmark, Norway, Sweden and Russia (54–56).

*Gymnopilus lepidotus*

This ground - dwelling macrofungi is a native of the tropical and sub-tropical forests. The convex cap is 0.4 -0.8 cm wide with the lamella that is placed on the adnated hymenium. Stipe is long, thick and has a tapering base. Spore print is mostly orange in color. With the few cases that has been reported so far it has been observed that this fungus may initiate severe allergic reaction in humans when consumed. The fungus is saprotrophic in nature and has been found to be reported earlier from Florida (United States), Argentina and Mexico(57,58).

*Gymnopilus purpureosquamulosus*

This ground – dwelling saprobic macrofungi is one of the rarest fungi that has been reported previously only from Zimbabwe, however later studies carried out to find its worldwide presence has reported its presence from Italy, Nigeria, Panama, and Switzerland. The convex pileus is 2.3 – 9.5cm wide which is raspberry pink in color when young and then becomes lilac colored with age. Lamella is crowded, close and is 0.4 – 0.8 cm wide. Stipe is cylindrical, thick with the length as 2.7 – 8 cm and width as 0.2 – 1.5cm. Spore prink is dark orange colored and the spores are ellipsoidal to oblong with the diameter as 6-8 µm (59–61). The surface ornamentation of the spore is warty with a conspicuous apiculus (Fig.4g).

*Gymnopus gibbosus*

This ground-dwelling fungus has been reported earlier from Australia (62). Cap is small and is mostly convex shaped. Stipe is long and polished. No other information could be accessed regarding its detailed appearance, habitat and distribution (63).

*Gymnopus luxurians*

This is a gregarious ground –dwelling macrofungi mostly found on the woodlands. Pileus is convex, 2-12cm wide with no mealy veils present on the top of the pileus with few brown colored streaks present on top. Lamella is attached to the stem and is pink colored. Stipe is 4-10cm long, 1cm thick with a bulbous base. Spore print is creamy white and the spores are smooth, lacrymoid to elliptical in shape with the diameter as 6.5-10 µm. It mostly appears when the conditions are humid (during fall/monsoon/rainy days) and has been reported earlier from eastern North America - Massachusetts, Alabama and Illinois (64,65).

*Gymnopus menehune*

This ground – dwelling fungus is commonly called as the ‘fungi of Hawaiian islands’. Dark brown colored stipe is convex shaped, hollow, 2.3 – 3.2 cm long and 0.8 – 3 cm wide. The fungus has a typical smell of a rancid radish. Lamella is crowded, adnate to sub-decurrent in arrangement. The spore print is pale orange in color and the spores are ellipsoidal, thin-walled, hyaline, and smooth, with the diameter of the spore lying between 3 - 4.2 µm. It has been reported earlier from Hawaii, Indonesia and Pakistan(62,66).

*Hymenagaricus epipastus*

Pileus of this ground – dwelling macrofungi is convex shaped and is 2cm wide. Stipe is 2 cm long and 0.17 cm thick. Spores are ellipsoidal in shape with the spore diameter lying between 3.5 – 4 µm. It has been reported earlier from Kerala and Sri Lanka (67).

*Hymenagaricus taiwanensis*

This ground – dwelling macrofungi, and all the other fungi belonging to the genus *Hymenagaricus* had been mostly reported from the tropical regions (58). It is a new species that has been reported only from Taiwan earlier (68). The pileus is yellow brown in color and has been covered by fuscous black squamules. The spores are ellipsoidal, 4 - 9 µm with a coarsely verrucosed surface (Fig.4h).

*Hymenogloea papyracea*

This fungus grows gregariously in the leaf litter of the tropical forests. It has been reported earlier from tropical America, Colombia and Venezuela. Cap is thin, convex and yellow colored. Stipe is long and slender. This macrofungi has no gills even though it belongs to the gilled macrofungi family (69).

*Lepiota atrodisca*

This ground – dwelling macrofungi has a yellowish white convex shaped pileus, 2.5–3.1 cm long with a dark black disc on the top. Lamella is free to crowded, yellowish white colored and is very fragile. Stipe is 7.8–8.6 cm long, 0.2–0.3 cm broad, yellowish white, hollow and scaly. Spore print is white colored and the spores are ellipsoidal, smooth, and thick-walled and have the dimensions as 5.5 – 8 µm. The habitat of this fungus is mixed hardwood forests; fruiting shortly after the rain (70). It has been reported earlier from California, Pacific coast of States, Santa Cruz and Himachal Pradesh of India (71).

*Lepiota castanea*

This ground – dwelling macrofungi is dangerously poisonous and is mostly referred by its common name, ‘chestnut dapperling’. The fungus is known to contain amatoxins which is known to cause lethal poisoning when consumed. The hairy pileus is umbonate and is 2-4 cm wide. Lamella is white colored but becomes brown with age. Stipe is scaly, 2.5 – 3.5 cm long and 0. 2 – 0.4 cm thick. Spore print is white colored and the spores are dextrinoid, bullet shaped with the diameter as 9 – 13 µm. It is found growing alone or gregariously in the deciduous and coniferous woodlands and has been reported widely from Europe (72,73).

*Lepiota Echinacea*

This ground – dwelling fungus is also called as the ‘dapperling’ fungi. The convex shaped cream colored pileus is 1.5-5 cm wide with pyramid shaped brown scales present at the top that are 0. 1 - 0. 2 cm tall. Lamella is free to crowded; initially it is pink color and becomes brown color with age. Stipe is 2 – 4 cm long and 0.25 - 0. 8 cm wide with a scaly base. Spore print is white colored and the spores are oblong to ellipsoidal, smooth with the diameter as 4 – 5 µm. It grows alone or gregariously in the mixed woodlands; however the occurrence has been reported to be very uncommon. The presence of this fungus has been reported earlier from Britain, Ireland and Italy (74,75).

*Lepiota ochraceofulva*

This ground – dwelling saprobic fungus is mostly found on the leaf litter. It is found in the spruce forests, beech forests and in the hedge rows. Pileus of the fungus is 2.8 -7 cm wide and the stipe is 5 – 7 cm long, 1cm thick. No information has been reported yet about the basidiospores of this fungus. This macrofungi has been reported earlier from United Kingdom, Sweden, and Germany (76).

*Lepiota phaeosticta*

This ground - dwelling macrofungi has a convex pileus that is 1-3cm wide with black scales present on the top. Lamella is free from stalk, crowded, finely fringed and doesn’t stain when bruised. Stipe is 3 - 6 cm long and 0.15 -0.3 cm thick with a clavate base. Spore print is white colored and the spores are 5 - 8 µm in size, ellipsoidal to ovoid and hyaline in nature. This is found growing alone or gregariously in the decaying woods and leaf litters. It has been reported earlier from Florida (77).

*Lepista densifolia*

This ground – dwelling macrofungi has a convex pileus that is 5cm wide and curved inwards at the margin. The fungus changes color from white to dark brown when damaged, because of the canescent layer getting disintegrated when pressed. Lamella is deeply decurrent, crowded, somewhat forked and beige colored. Stipe is 8 – 10 cm long, 0.5-1.5 cm thick, hollow and canescent. Spores are broadly ellipsoidal with a conspicuous apiculus and has the diameter as 4 - 4.5 µm. It appears gregariously in the grasslands and woodlands and has been reported earlier from Norway and Central European mountains (78).

*Lepista flaccida*

This macrofungi is popularly known by its synonym, ‘*Clitocybe flaccida*’. One distinctive feature of this fungus is its exact funnel shape, which makes its identification easy. The convex shaped pileus is 4 - 9cm wide, funnel shaped with a wavy in-rolled margin. Initially the pileus is orange – brown in color as the fungi is hygraphanous in nature, the color becomes pale as the pileus starts drying – off. Lamella is deeply decurrent, crowded, white colored when young and becomes tawny with age. Stipe is 3 - 5 cm long and 0.5 - 1cm thick with no stem ring. Spore print is creamy white in color and the spores are broadly ellipsoidal, minutely warty with the diameter as 4 - 5 µm. It occurs in all kind of woodlands and has been reported earlier from mainland Europe and North America (75,79).

*Lepista nuda*

This macrofungi is mostly recognized by its synonym, ‘*Clitocybe nuda’* and it is also known by its common name, ‘blewit’. The fungus initially adorns shades of lilac and blue but it fades away very quickly that it becomes brown in color. This is a ‘bacterium destroying basidiomycota’ (80). The tiny hyphae sent out by this fungus, penetrate the bacterium colonies, and kill them by sucking out their nutrients. The convex to flat pileus is 4 – 20 cm wide. Lamella is attached to the stem and is crowded. Stipe is 3 -10 cm long and 1-3cm thick with a bulbous base. Spore print is pale pinkish and the spores are elliptical, minutely roughened with the diameter as 6 - 8 µm. This fungus occurs alone or gregariously in the hard woodlands and has been reported widely from North America (81–84).

*Leucoagaricus atrodisca*

This is commonly called as the ‘parasol mushroom’. This fungus contains highly deadly toxins as the *Amanita phalloides* (death cap). The symptoms usually develop from 6 to 15 hours of ingestion. There is no known antidote available for this poisoning till date. The convex shaped pileus is 1.5 - 4cm wide with a curved margin. White colored lamella is free and closed with fringed edges. Stipe is 2 - 8.5cm long, 0.1 – 0.4 cm thick with a membranous veil. Spores are ellipsoidal, smooth, relatively thick walled and the diameter is 4 - 6 µm (Fig.4i). It is found growing alone or gregariously in the mixed hard woodlands and has been reported earlier from California (85).

*Leucoagaricus bresadolae*

This macrofungi is also called as *Lepiota bresadolae* and is suspected to be poisonous*.* The convex to flat pileus is 3 – 8 cm wide, white, umblicate at center and is covered by reddish brown scales. The flesh of the entire fruiting body is thick and changes its color from white to red when bruised. Lamella is free and crowded and is creamy to white colored. Stipe is 5.5-11cm long, 0.4-1cm thick, cylindrical, hollow and has got brownish scales at the bottom. Spore print is yellow and the spores are broadly elliptical with conspicuous apiculus, thick walled and smooth with the diameter as 9 - 11 μm. It is found growing gregariously in the grasslands, broad-leaved forests and has been reported earlier from Taiwan and Japan (86,87).

*Leucoagaricus cepaestipes*

This is also called as *Leucocoprinus cepaestipes* and the common name for this fungus is ‘onion – stalk Lepiota’. The bell-shaped to convex pileus is 2 - 6.5 cm wide. The surface of the pileus is granular to scaly and has a pinkish tan to grayish brown disc at the top. Stipe is 4 -11.5 cm long with a membranous veil present at the top and a bulbous base at the bottom. Stipe changes its color from pale pink to pale yellow when bruised. Spore print is white and the spores have the diameter as 8 -10 μm, ellipsoidal in shape with a conspicuous apiculus. This fungus occurs gregariously in the rich soil of woodlands and has been found widely distributed in North America, Europe and is common in the Eastern parts of the world. This fungus is highly likely to be poisonous (88–90).

*Leucoagaricus meleagris*

This ground – dwelling macrofungi is also called as the Psalliota meleagris. Pileus of this fungus is 5 -14 cm wide, plano-convex to applanate, with a depressed center and top of the pileus is covered by brown to grey squamules. The white colored fruiting body turns yellow when rubbed mildly, and the color becomes brown with time. Lamella is crowded and is brown to black in color when fully mature. Stipe is 5 -14 cm long, 0.7 – 1.8 cm thick, clavate to cylindrical with a bulbous base. Spores are oblong to ellipsoidal with an inconspicuous apiculus having diameter as 4.5 - 6.5 μm. It is mostly found in mixed woodland and has been reported earlier from North America, Britain and Europe (20,91).

*Leucoagaricus rubrotinctus*

This fungus is commonly called as the red-tinged Lepiota as it has a very distinct coral pink color when young. The conical pileus is 3 – 8 cm wide, initially the fungus is egg-shaped, and with the age the pileus becomes flat. Lamella is not attached to stem, closely packed and doesn’t stain when bruised. Stipe is 4 -16 cm long and 0.5 – 1 cm thick with a bulbous base. Spore print is white colored and the spores are ovoid to elliptical in shape with the diameter as 6-10 µm. This saprobic fungus is mostly found in the woodlands and has been reported to be widely present in North America (92,93).

*Leucoagaricus tangerinus*

This macrofungi has two distinctive features: the presence of orange brown thick squamules on the top of the pileus and the presence of colorless droplets in the white stipe. Pileus is convex shaped and is 2 -5 cm wide. Lamella is free from stem and is moderately crowded. Stipe is 6.5 – 7 cm long, 0.3 – 0.4 cm thick, cylindrical in shape with a bulbous base. Spores are ellipsoidal to ovoid with the diameter as 6.5-7 µm, smooth, slightly thick walled and have a fairly visible apiculus. The saprobic fungus is mostly found in broad-leaved forests and has been reported earlier only from China (94).

*Leucoagaricus vassiljevae*

This macrofungi is recognized by the reddish tinge of its basidiocarp. Pileus is 1.5 - 4.5 cm wide, convex shaped and is covered with reddish –brown fibrils on the top. Lamella is not attached to stem, crowded and almost vertical. Stipe is 5 – 13 cm long, 0.2 - 0.5cm wide, uniformly thick with a bulbous base. Basidiospores are oblong to ellipsoidal with a conspicuous apiculus and having the diameter as 8 - 12 µm. It is found solitarily in the leaf litters of coniferous and deciduous forests. It has been reported earlier from Russia and Gujarat, India (31).

*Marasmiellus palmivorus*

This fungus is a potential pathogen causing bunch rot disease in oil palm fruits, seeds and seedling. Pileus is 0.4 – 3 cm wide, plano-convex shaped, striated and has an incurved margin. Stipe is 0.4 – 1.7 cm long with a tapering base. Lamella is free, adnexed and periodically furcates. Spore print is cream to orange brown in color. No information is available about the size and shape of the spores. This fungus mostly occurs in the tropical regions and has been reported earlier from Malaysia, Indonesia and Hawaii (95,96).

*Marasmius albimyceliosus*

Pileus is 1.5 – 3.8 cm wide, plano – convex when young and becomes plano – concave when mature, glabrous, striated and is light brown in color. Lamella is adnate, narrow, cream colored, non – marginate and poroid – reticulate. Stipe is 2.3 – 5.3 cm long, 0.1 – 4 cm thick, glabrous, cylindrical with a sub-bulbous base. Spores 6 - 8 µm in diameter, ellipsoidal, smooth, hyaline, and thin- walled. This ground – dwelling fungus has been reported earlier from Thailand and Singapore (97).

*Marasmius haematocephalus*

The strongly red colored pileus of this macrofungi is 0.3 – 1.5 cm broad, striated, convex shaped with an uplifted margin. Lamella is free to adnexed, narrow to broad, white, pale purplish, with concolorous pubescent edge. Stipe is 2 – 5 cm long, 0.2 - 0.5 cm thick, cylindrical, hollow, glabrous and dark brown colored. Spores are 3.5 – 5 μm in diameter, clavate-fusoid, thin-walled and hyaline. This fungus is widely found alone or gregariously in the grasslands and is widely distributed in Africa, Northern Thailand, Malaysia, China, Kerala (India) , Ceylon, Surinam, Brazil, Guiana, Cuba and United States (62,97–99).

*Marasmius nigrodiscus*

This is uncharacteristically large for the genus *Marasmius*, with a cap that can reach a width of 11 centimeters. The pale brown pileus is 3-11cm wide and plano-convex or nearly flat in shape with a central bump. Lamella is broadly or narrowly attached to the stem, white to grey in color. Stipe is 4 – 15 cm long, 1 cm thick, straight, bald or finely silky with shallow longitudinal grooves. Spore print is white colored and the spores are elliptical with the diameter as 7 - 9 µm. This saprobic fungus is mostly found on the litter of the hardwood forests and has been reported earlier from North America (100,101).

*Marasmius nummularius*

The reddish brown pileus of this macrofungi is convex shaped and is 0.5 - 1.6 cm wide. Lamella is adnate, crowded and yellowish white in color. The cylindrical orange – brown stipe is 1.6 – 4 cm long and 0.1 cm thick. Spores are 4 - 4.5 µm in diameter, elongated to fusoid in shape. It is found solitarily or gregariously on the leaf litter of grasslands and has been reported earlier from Malaysia and North American mountains. It has been stated as one of the threatened species in Thailand (102,103).

*Marasmius occultatiformis*

Pileus of this fungi is 8-15mm wide, hemispherical to convex shaped when young, planar when mature. Center of the pileus is pitted, margin inflexed, translucently striated and is deep orange in color. Lamella is toothed, moderately toothed, whitish cream in color with an orange color edge. Stipe is 2.5 – 4.5 cm long, 0.1 – 0.2 cm thick, cylindrical, slightly broadened downward, cartilaginous, glabrous, whitish at the top and reddish brown to dark brown at the bottom. Spores are 6.5 – 11 µm in diameter, smooth, ellipsoidal to fusoid and thin-walled. It is mostly found on the leaf litter of mixed forests and has been reported earlier from Russia, Siberia and Korea (104).

*Marasmius ochroleucus*

Pileus is 0.6 – 1.5 cm wide, hemispherical to convex in shape, slightly uneven, hygrophanous with the margins having yellowish tint and translucent striation. Lamella is adnexed, crowded, thin and white in color. Stipe is 30 – 35 cm long, 0.15 – 0.2 cm thick with a cylindrical and bulbous base. Spores are 8 – 10 µm in diameter and ellipsoidal in shape. It is mostly found either alone or gregariously on the leaf litter of mixed forests and has been reported earlier from Russia, New Caledonia and Northern Thailand (104).

*Marasmius oreades*

The common name of this fungus is’ fairy ring mushroom’. Apart from the ability to form fairy rings, this fungus has the unique capability of reviving itself after complete dryness unlike the other macrofungi. This is because of the presence of trehalose; a type of sugar when exposed to water enables the fungi to revive itself by cellular reproduction and creation of new spores (105). Pileus is convex shaped and is 1 – 5 cm wide. Lamella is nearly attached to stem or free from it, distantly placed and white in color. Stipe is 2 -8 cm long, 0.15 – 0.6 cm thick, dry and tough. Spore print is white colored and the spores are 7-10 µm in diameter, smooth and fusoid-ellipsoidal in shape. The saprobic macrofungi grows in grasslands and also in the coastal grasses in dunes. It has been reported earlier from North America and Europe (106).

*Marasmius trichotus*

This yellow to orange colored fungus has its pileus, which is 0.6 – 2.3 cm wide, convexly shaped when young and then becoming planar with age. Lamella is adnate and closely packed. The orange to red colored stipe is 1.5 – 9 cm long, 0.5 – 1 cm thick, cylindrical and hollow. Spores are 10 - 15 µm in diameter, narrowly ellipsoidal, hyaline and thin-walled. It is found either scattered or gregarious in the grasslands and has been reported earlier from Northern Thailand, Papua New Guinea and Singapore (102).

*Micropsalliota alba*

The cream colored pileus of this macrofungi is 0.4 – 0.8 cm wide, 0.4 – 0.6 cm tall, conical in shape, striated with a partial veil. Lamella is free from stem, crowded, ventricose and light brown in color. Stipe is 2 – 2.5 cm long, 0.6mm thick, cylindrical, hollow, smooth and white in color. The fruiting body stains reddish brown when bruised. Spores are 5.5-7 µm in diameter, elliptical in shape with an inconspicuous apiculus. This fungus grows gregariously in shaded area where there is high humidity. It has been reported earlier from Thailand and Kerala, India (107).

*Micropsalliota globocystis*

The reddish brown pileus of this fungus is 1.7 – 6 cm wide and is plano-convex and umbonate in shape. Lamella is free from stem, crowded and is orange- grey in color. Stipe is 4 -12 cm long, 0.3 – 1.2 cm thick, smooth, cylindrical and is reddish brown in color. Spores are 5-10 µm in diameter, ellipsoidal and hyaline (Fig.4j). It is found growing solitarily or gregariously in rich soil. It has been reported earlier from Singapore, Thailand and Kerala, India (107).

*Micropsalliota lateritia*

Pileus is 0.1 – 1.8 cm wide and plano-convex in shape. Lamella is free from stem and closely packed. Stipe is 1.5-1.8 cm long, 0.15 cm thick, cylindrical, hollow, smooth and violet red in color. Spores are 5 - 6 µm in diameter, ellipsoidal, thick- walled with no apiculus. It is found growing scattered in the forests and has been reported from Thailand (107).

*Omphalotus japonicus*

This orange to brown-colored poisonous fungus grows only on trees and its half-moon or kidney shaped pileus is 2 cm (0.8 in) thick. The thick and fleshy stem is 2 cm thick and 5 cm long. It has been reported that the spores of this fungus is gigantic compared to the other species of this genus. The fruit body has the property of bioluminescence. The fungus is found growing on deadwoods and is widely spread in Japan and Eastern Asia (108,109) .

*Omphalotus olivascens*

This is orange to brown-colored gilled mushroom found growing only on trees is commonly known as the ‘western jack-o'-lantern mushroom’. This fungus is said to be toxic, yet not lethal. The cap is smooth, convex to flat at first and then points up with age. Stipe is 5 – 15 cm long, 1 – 4 cm thick, tapering downwards and smooth textured with brownish stains. Gills are decurrent and luminescent. Spore print is cream to pale yellow colored and the spores are 6.5 – 8 µm in diameter, globose to ovoid, smooth and non-amyloid. This gregarious and saprotrophic fungi found growing mainly on the oak trees has been reported earlier from California and Mexico (111).

*Omphalotus subilludens*

This is an orange colored poisonous fungus with conspicuous decurrent lamellae. Gills of the fungus are known to have the bioluminescent property. The spore print is cream-colored and the spores are 6 - 10 µm in diameter, narrowly ellipsoidal, hyaline, and thin-walled. This gregarious and saprotrophic fungus grows mainly on oak and other hardwoods. It has been widely reported to be present in Europe, North and Central America (110,112).

*Paralepista gilva*

This saprobic fungus is one of the rarest and toxic ground dwelling agaric. The fungus possesses a fleshy basidiomata, lamellae that is adnate to decurrent and a convex to funnel-shaped pileus. The spore print is whitish to pinkish yellow and the spores are smooth and non-amyloid (113). It is found to be growing scattered or gregarious on pinaceous needle-beds and debris, often together with *Lepista flaccida*, on calcareous soil. The fungus been reported earlier only from Morocco, France, Spain, and Italy (113).

*Parasola auricoma*

This is one of the small, short- lived gregarious and saprotrophic agaric found growing on grasslands and woodlands. The fungi has an umbrella shaped fruiting body which is initially egg-shaped with margins curled inward; as the cap expands, it becomes conical and eventually flat reaching a diameter of 6 cm . The fruit bodies are hydrophanous and thus changes color depending on their state of hydration. The lamellae are free from stem and are 0.2–0.4 cm wide. The stipe is 3.5 - 12 cm long, 0.3 cm thick, fragile, silky and whitish yellow in color. Spores 10-16 µm in diameter, dark brown, smooth, ellipsoidal and thick-walled with a conspicuous apiculus. It has been reported earlier from Europe, North America and Hawaii (46,114).

*Pholiota spumosa*

This gregarious and scaly agaric is a small to medium sized fleshy fungi typically found in the woodland. Pileus of the fungi is glutinous to dry and scaly in nature. The scaly feature of the pilei is the evolutionary change that the fungus has adopted to avoid the feeding of insects on the tissues of the fungi. The spore print is reddish-brown in color and the pores are elliptical to oblong-elliptical in shape with diameter as 6 - 9.5 µm (Fig.4k). It has been reported earlier from Canada (115).

*Pleurotus djamor*

This is commonly referred as the ‘pink oyster mushroom’ and is mostly found in the tropical woodlands. The pileus is fan-shaped and is 2 to 5 cm wide. The lamella is strongly pigmented with pinkish tones when young, fading to creamy beige with age. Spores are 6 - 10 µm in diameter, smooth, and cylindrical with a conspicuous apiculus. This edible gregarious fungus has been reported in Bihar, India where it was found to occur widely in the month of October (116).

*Pluteus petasatus*

This white colored edible fungus has its pileus which is 5 -13.5 cm wide, convex to convex-umbonate when young and becoming planar with age. The surface of the pileus gets sticky during moist conditions. The lamellae are crowded and the cream color changes to salmon pink when mature. The stipe is 5-9 cm long and straight. The spore print is salmon-pink and the spores are 5.5 - 7 µm in diameter, broadly ellipsoid to ovoid and smooth. This gregarious fungus grows in the woodlands on rotting wood and is often spotted after the rains. It has been reported earlier from North America (117,118).

*Pluteus romelii*

Cap is umbonte and convex that gets flat with age. Gills are free from stipe. The *Pluteus* species generally lack a volva. The spore print is pink and the spores are smooth and egg-shaped. The fungus has been reported to have neurotropic (hallucinogenic or psychotropic) properties. It is commonly found in the woodlands, growing on the rotten woods closely towards the ground. Some of the species belonging to this genus have been reported from Europe and North America (119).

*Pluteus thomsonii*

This is generally called as the ‘veined cap’ fungus and is generally distinguished from other species of this genus by its diminutive size. The blackish brown pileus is 1-3.5 cm wide and is convex shaped that becomes broadly convex with age. The gills of the lamellae turn white to pink color with age. The silky streaked stipe is 2 - 4.5 cm long and 0.15 -0.6 cm thick. The spore print is pink and the spores are 6-8 µm in diameter, elliptical and smooth. This gregariously growing fungus is known to occur in the woodlands and has been reported from eastern North America, and documented in California (119).

*Psathyrella candolleana*

This white to golden brown poisonous fungi is an inhabitant of the grasslands and woodlands; found growing alone or gregariously. This was one of the most abundantly found species in the study region. The pileus is rounded – conical or convex in shape with potassium hydroxide present on its surface. The crowded gills are attached to the stem and color changes from honey yellow to darkish brown with age. The delicate stipe is 4 -13 cm long and 0.3 – 0.8 cm thick. The spore print is dark purplish color and the spores are 6.5 - 9.5 µm in diameter, smooth and ellipsoidal with a truncated end. SEM images were taken for both young and mature spores and the images are given in the Fig.4l and Fig.4m. This fungus has been widely reported from Europe, North America and Iraq (120).

*Psathyrella gracilis*

This has been reported to be one of the most slender and fragile species belonging to this genus. The pileus is hydrophanous and becomes pink with age; also the cap (conical, 1.5-3.5 cm wide) develops striation when moist. Lamella has two-tiers of gills that are closely packed. The stipe is found to be longitudinally striate. The spore print is dark chocolate brown or purplish brown and the spores are 5-5.5 µm in diameter, elliptical and smooth and with a distinct apiculus (Fig.4n). Solitary yet mostly gregarious; found growing on the grassland and woodlands mainly during the rains. It has been reported earlier from New Zealand, North America and Europe (121,122).

*Schizophyllum commune*

This ‘split-gill’ fungi is a very common species that is found globally yet mainly in the tropic regions. It is generally found in the rotting wood of the hot and humid tropical region. The cap is shell-shaped and lobed, 1 - 5 cm wide. Gills are greyish-white in color and are 4 cm wide. The spore print is white and the spores are 3 - 4 µm in diameter, smooth and cylindrical to ellipsoidal in shape. It has been reported to cause moderate to severe (in immunosuppressed patients) respiratory allergies to humans when inhaled. Its abundant presence has been reported from north-eastern India mainly in Mizoram, where it is consumed as a delicacy. It has been reported earlier from North America and throughout the world (123).

*Scytinopogon angulisporus*

This highly branched fungus is commonly referred as the ‘finger sponges’ that is 30 mm in height. The stipe is white to cream colored and is poorly differentiated. Spore print is white and the spores are echinulate or verrucose and angular. This highly gregarious and terrestrial species is mainly found in the tropic region. It has been reported earlier from Mexico and West Bengal, India (124).

*Termitomyces heimii*

This edible and therapeutic fungus is one of the most predominantly present agaric in India especially in the south India. The agaric has a symbiotic relationship with the termites and thus it gets the name. The pileus can extend up to 10cm in diameter and is convex in shape. Lamella is generally white to pink colored. The stipe is smooth, cylindrical and ranges from 1.5 – 19 cm. The spore print is pink in color and the spores are 7 - 8.5 µm in diameter, ellipsoidal and smooth. These basidiomycetes are distributed throughout tropical and subtropical areas of Africa and Asia (125).

*Termitomyces microcarpus*

This gregarious, edible agaric has also got a symbiotic relationship with the termites, especially the ones found near the bamboo stumps. This species has been reported to be predominantly present in the evergreen forests. This is a medium sized fleshy agaric with a whitish cap and a whitish gill. Pileus is 2 – 5 cm wide with a smooth to silky texture and the stipe is cylindrical. Spores are ovoid to ellipsoidal, 6-7 µm in diameter. It has been reported earlier from Africa and India. In India it has been reported from Goa, Karnataka and in the south-western region of the Western Ghats (126).

*Tricholoma mongolicum*

This is edible – medicinal basidiomycetes with a sinuate lamella. Cap is broad; convex shaped but flattens with age. Stipe is long and even throughout the base. The spore print is pale white in colour. It is solitary, saprotrophic, ectomycorrhizal and is mainly found in the grasslands with its presence being reported from North China and Mongolia (127).

*Volvariella dunensis*

This ground dwelling agaric is found mostly in the dry ecological habitat. The basidiocarp of the agaric grows deeply buried in the ground. The sub-globose brown to grayish brown pileus is 3.5 – 10 cm wide. The white colored cylindrical stipe is 2cm long. The basidiospores are 7 - 8.5 µm in diameter and are ellipsoidal to oblong in shape. It has been reported earlier from two localities on the Mediterranean coast of Spain (128).

*Volvariella hypopitys*

This is a ground dwelling edible agaric found mostly in the woodlands and grasslands, growing gregariously or in solitude. The pileus is 2 - 6 cm wide and is bell shaped or convex in shape. The white colored gills are minutely fringed. The stipe is 2 – 8 cm long and is slightly tapered towards the apex (129).

*Volvariella taylorii*

This edible agaric is generally found in the decayed woods of the woodlands. The pileus is 2 – 6 cm wide and is conic to convex in shape. The lamella is 0.4 -0.7 cm broad and is whitish to salmon colored. The stipe is solid, round, white colored with the following size 3.5 – 6.5 cm x 0.3 – 0.7 cm. The spores are dark rose, 5 - 9 µm in diameter, elliptic and smooth (Fig.4o). It has been reported earlier from Pacific North West (130).

*POLYPORALES*

*Ceriporia lacerata*

This is a wood-inhabiting polypore that produces resupinate basidiocarp and causes white rot in trees. The fungus has got variable colors of poroid surface, a monomitic hyphal structure with simple septa on generative hyphae. This fungus has been known to cause a wide spectrum of clinical manifestations ranging from saprobic colonization to fungal pneumonia in patients with respiratory diseases. Spores are 5-5.5 μm in diameter, elliptical to oblong in shape with a conspicuous apiculus (Fig.4p). It has been reported earlier from Europe, North and South America, Australia and U.K (131,132).

*Coriolopsis byrsina*

This is a wood dwelling fungus found mainly on the rotten woods. The basidiocarps are annual, resupinate, brittle when dry, up to 8.3 cm long, 5.4 cm wide, and 1.7 cm thick at center. The hyphal system is monomitic. No information is available about the basidiospores. It has been reported earlier from tropical regions (16) and east Africa (133).

*Coriolopsis caperata*

The fungus found attached to the trees has a fan shaped cap. Also the caps are tough and poroid in nature. The fungus is said to be a potential plant pathogen. It has been reported earlier from North America (16).

*Coriolopsis gallica*

This fungus is said to be saprobic causing white rot in the tree that it inhabits. The cap is usually present and well developed, but sometimes it is found to be folded-over edge above a spreading pore surface; up to about 20 cm wide, 10 cm deep near the base, convex; semicircular and bracket-shaped. The cap is densely hairy, becomes bald on the margin upon maturation. Spores are extremely variable in size, even within a single fungus. It has been reported earlier from North America (134).

*Dichomitus squalens*

This is commonly referred as the red rot fungus, causing extensive decay even in young trees. The fungus has a flat fruiting body and is usually present in the underside of the tree branches. The pore surface is white colored when young and turns yellow with maturation. The fungus has been reported earlier from south west America (135).

*Earliella scabrosa*

This tough tropic polypore is a known white rot fungus. This is a monotypic genus containing only this species. Basidiocarp is resupinate, shell-like and pileated. At first it is white to cream color, soon covered by a reddish cuticle starting from the base which is 1 cm thick. Basidiospores are 8 - 12.5 μm in diameter and are cylindrical to oblong in shape. It has been reported earlier from Taiwan (136).

*Favolus emericii*

This polypore is known to cause white rot growing as a parasite on living trees. Cap is fan shaped and broadly convexed. The pore surface is white colored and irregular. Stipe is short, thick and placed off-central. The fungus is reported to be distributed in Philippines, Ceylon, Australia, New Zealand, Cuba, New Guinea and Brazil (137).

*Flavodon flavus*

This marine basidiomycete is reported as the white rot lignin-modifying polypore. This has a resupinate basidiocarp that is firmly attached to the woody surface of the trees. The pore surface is irregular. This fungus is capable of degrading azo dyes (138).

*Fomes fomentarius*

This plant pathogenic fungus, also called as the ‘horse hoof’ is known to cause rots in trees. They continue to grow on dead trees changing their role from parasite to decomposer. This perennial polypore has it cap extending up to 20cm. The pore surface is brownish with 2-5 round pores per mm. This solitary fungus generally prefers hot climatic conditions for its growth and has been reported earlier from Europe, Asia, Africa and North America (139).

*Ganoderma applanatum*

This wood decaying fungus is also called as the ‘artist’s bracket’. This cosmopolitan fungus is normally seen at the stump of the trees. The brownish to grayish brown cap is 5 – 75 cm wide with a dull, unvarnished outer crust; often furrowed in "zones". The pore surface is white to brown in color. Spores are brownish red in color, 8 - 12 µm in diameter; more or less elliptical, with a truncated end. This fungus has been reported quite commonly from England and United states (140,141).

*Ganoderma carnosum*

This fungus is morphologically similar to *Ganoderma lucidum*. It has been reported to be present in the hardwoods mainly on the fir trees. Unlike other Ganoderma species, a stem is present in this fungus and the brown to yellow cap is shiny and zoned. Spores are 11-13.5 µm in diameter and this fungus has been reported earlier from Europe (142).

*Ganoderma lucidum*

This oriental fungus also called as the ‘Reishi mushroom’ is well known for its medicinal purposes. This fungus is distinguished from other species by its varnished red cap. It has been reported that this saprophytic fungi tends to grow more prolifically in the warm climate on the decaying hardwood, logs and stumps. Stems are non-existent for this species. Spores are generally brown in color, ellipsoidal and is 8 – 6 µm in diameter (Fig.4q). It has reported earlier from Eastern countries and many Asian countries (143,144).

*Ganoderma multipileum*

This species is highly similar to *Ganoderma lucidum*. It is also known to have medicinal properties. The medicinal usage of this fungi dates back to 2000 years. This saprophytic fungus is known to grow on dead hardwoods. The cap surface is dark brown in color. The pore surface is white and the spores are brown in color. It is widely found in the tropical Asian countries as it grows abundantly in the warm climate(145).

*Ganoderma neojaponicum*

This saprobic shelf fungus is found in Mainland China, Japan and Taiwan growing on dead hardwoods or bamboos. This fungus is reported to have anti-tumor and anti-hepatotoxic activity. The cap is dark reddish brown in color and the pore surface is white in color like the other *Ganoderma* species (146).

*Ganoderma tsugae*

This flat polypore is also called as the ‘Hemlock varnish shelf’. This fungus is closely related to *G. lucidum*; however *G.tsugae* tends to grow on conifers. Pileus is 5 -30 cm wide, irregularly knobby with a lumpy varnished surface. The fruiting body is reddish brown in color when mature; while young it is zoned and is bright yellow in color. The pore surfaces are white when young and brown as it matures. Stem is 3 -14 cm long, twisted and irregular. Spores are 13 - 15 µm in diameter, brown colored and ellipsoidal with a truncated end. This macrofungi has got anti-tumor activity and has been reported earlier from China and New Mexico (147,148).

*Lenzites elegans*

This annual to perennial polypore is a reported plant pathogen causing white rot in hardwoods. The cap is 1 - 35 cm broad, 0.2-3 cm thick, flabelliform, dimidiate, circular, sessile, concentrically sulcate and warted. The texture of the fungi is corky, flexible when fresh and more rigid as it matures. The pore surface is white and the pores are very variable. The spores are 5 - 7 µm in diameter, oblong, ellipsoidal, and hyaline. It has been reported earlier from Southeastern United States, also it’s widespread presence has been reported over all the tropical regions (149).

*Lopharia cinerascens*

This polypore is said to be cosmopolitan in distribution. The cap is gray colored, effused and coriaceous. The pore surface is irregularly and shallowly poroid with the basidiospores having the diameter as 8 - 14 µm. It has been reported earlier from United States, Canada, Cuba, Jamaica, Mexico, Venezuela and South Africa (150,151).

*Microporus ochrotinctus*

This polypore has been reported earlier from Pacific Islands. This tree dwelling fungus is known to grow mostly on the deadwoods. Cap is fan shaped and the outer surface is ridged. Stem is short and thin, found protruding from the tree surface where it is attached. The pore surface is white and even (152).

*Perenniporia fraxinea*

This white rot fungus is known to attack the trees by degrading the lignin resulting in the fracture of the affected region. The fungal cap has a flattened upper surface with a knobby texture that is almost warty. They are said to have a symbiotic relationship with algae that results in a green color cap. The fruitbodies of *P. fraxinea* have one unique identification feature: when cut open, the corky textured flesh is the same creamy fawn color as the (fresh) exterior. This uniformity of coloration contrasts well with species of the Ganoderma genus. The basidiospores are white colored with a talcum powder texture. It has been reported earlier from Northern and Central Europe (153).

*Pereniporia ochroleuca*

This perennial polypore is also known to cause white rots in the trees. The fungal cap is grayish black with a corky texture and a pale ochraceous upper surface. The cap is 7cm wide and 1 - 2.5 cm thick. The pore surface is white colored and the spores are ellipsoidal, 12 - 17 µm in diameter, truncate at the apex, thick walled and smooth. It has been reported earlier from Madeira island of Portugal and China (154).

*Polyporus grammocephalus*

This fungus is common to Indian hardwood forests. The stemmed basidiocarp is annual, solitary, and laterally stipitate. The reddish brown cap is fan shaped, ovate or reniform, 9 – 12 cm long, 6 - 9 cm wide, 0.1 – 0.3 cm thick with an upper surface that is glaborous and striated. The spores are 5 - 7.5 µm in diameter, cylindrical, hyaline and thin walled. It has been widely reported from West Bengal, India (137).

*Trametes elegans*

This inedible saprobic polypore is found growing either solitarily or gregariously on the dead and decayed hardwoods. The white to grayish caps are 2 -14 cm wide, glabrous, warted and concentrically grooved. The pore surface is whitish and is either gill-like or maze-like. Stem is absent or rudimentary. The basidiospores are 5 - 7 µm in diameter, white, smooth, and elliptical. It has been reported that this fungi is widely found in the tropical regions and its presence has been reported from Nigerian forests (81,155).

*Trametes robiniophila*

This polypore has been reported to have anti-tumor activity over the human hepato-gastric carcinoma cells. The fungus is known to cause white rot in hardwood forests. The fungus has its appearance similar to that of the other *Trametes* species (164).

*Trametes suaveolens*

This inedible fragrant saprobe found growing on the dead broad-leaved hardwoods is also called as the ‘lumpy bracket fungi’. It is reported to have a symbiotic relationship with the algae causing to have a greenish tinge on the upper surface of the cap. The cap is 5 – 16 cm wide, 1 - 10 cm long and 1 – 4 cm thick, the upper cap surface is white, hairy to glabrous and somewhat watery when fresh. The pore surface is whitish to yellow with age. This fungus is known to possess lignin – degrading capability. It has been reported from United Kingdom and Netherlands (157).

*Truncospora macrospora*

This polypore is known to have a distinct annual habit, found growing on the hardwood. The basidiocarp is found pileated. The spores are 8 – 9.5 μm in diameter and dextrinoid. It has been reported widely from South west China (158).

*Truncospora ochroleuca*

This perennial saprobe is found to have a corky basidiocarp. The pileus is glabrous, concentrically zoned and resinous. The pores of the poroid surface found underneath are obscurely stratose or continuous. The spores are 12 - 16 µm in diameter, narrowly elliptical or elliptic-obovate, apices rounded, bases truncate walls smooth and hyaline. The presence of this fungus has been reported earlier from Africa, London, Ceylon, Japan, Australia, Tasmania and New Zealand (159).

*Hymenochaetales*

*Fulvifomes fastuosus*

This solitary tree dwelling fungus is mostly found on the tree stumps growing solitarily. The pileus is applanate, brown, woody hard projecting upto 15cm, 13.5 cm wide and 2 cm thick at the base. The pore surface is umber brown and circular. Basidiospores are sub-globose, thick walled, yellowish brown and smooth with the dimensions of 4.49 μm as mean spore width. It had been reported as one of the novel species from Sri Lanka. It has also been reported from West Pakistan and Tokyo (160,161).

*Phellinus repandus*

This is a well-known tree rot fungi. *P. repandus* attacks the wood by forming small longitudinal whitish pockets which eventually makes the wood fragile resulting in the crumpling of the wood. The distinguishing feature of this fungus from other rot causing fungi is that, *P.repandus* can attack live trees unlike the other rot fungi that attack only dead woods. Basidiocarp appears greyish brown with a bright yellow margin. The pore surface texture is that of a sand paper. The basidiospores are 3 - 4 µm in diameter and ovoid to sub-globose (Fig.4r). It has been reported to be widely present in the Western United states (162).

*Pyrrhoderma scaurum*

Basidiocarp of these annual fungi is pileate, dimidiate to flabelliform or laterally stipitate, up to 10 cm long and wide, 0.8 cm thick at the base, dense and woody mostly. The pore surface is yellowish brown to umber, pores angular to circular with pores every 4 - 5 per mm. Basidiospores are 5 - 6 µm in diameter, globose to subglobose, thin-walled and hyaline. This hardwood fungus is generally known to have an Asian origin. It has been reported earlier from temperate China (Zhejiang), Japan (Honshu, Hokkaido), and Far East Russia (163).

*Geastrales*

*Geastrum pectinatum*

This highly rare humanoid fungus is also known as the ‘Beaked earthstar’. It is difficult to locate this fungus on the ground as it blends easily with the fallen leaves. Geastrums are characterized by a significant spore sac, ray structure and a peridium. Spore sac is pale greyish-blue to greyish- violet, 1-3 cm across and mostly subglobose. The beak of the spore sac sits in a shallow depression. The spore sac is situated above its base which is separated by a short stalk that has no basal collar - this feature differentiates the *G.pectinatum* from the very similar Geastrum striatum, which has a basal collar. Spores are globose, warty, 3-4 µm in diameter (Fig.4s). It is found to occur in the hardwood forests and it has been reported widely earlier from London and Ireland (164,165).

*Geastrum striatum*

This inedible fungus is also known as the ‘Striated earthstar’. This fungus is not often recorded but known since 1805. The spore sac is dull greyish-brown coming to a distinct point at the apex, sitting on top of a more brownish, star-shaped base with 6 to 9 rays. Basidiospores are spherical, warty and are 3 – 4 μm in diameter (Fig.4t). These are widely seen growing in the sandy soil and tend to grow under the trees. They often occur during the summer and autumn. It has been reported earlier from Leicestershire and Rutland (166).

*HETEROBASIDIOMYCETES*

*Auriculariales*

*Auricularia fuscosuccinea*

This belongs to the genus that is commonly called as the ‘Jelly fungi’. No specific information is available for this particular species, yet the fruiting body of all the species belonging to the genus *Auricularia* share few common features. The fruitbody is either resupinate or pileate, ear-to shell-shaped or bracket shaped, flabby elastic or tough gelatinous. Spores are narrowly ellipsoid to allantoid, hyaline, smooth. This has been widely reported to be present in the Western Ghats of Kerala (167).

*Auricularia polytricha*

This edible jelly fungus is commonly called as the ‘Cloud ear fungi’. This is a well-known Chinese medicine. The high content of polysaccharides makes them highly edible. Fruiting body is as said ear shaped, medium sized, dull in texture, and dark brown to black. The lower surface is wavy and has a contrasting powdery gray color. The gills and stem is absent. Basidiospores are 13 – 16 μm in diameter, hyaline, reniform to allantoid and guttulate. This fungus is widely found growing in the deciduous to evergreen forests and has been reported widely to be present in the Western Ghats of Kerala (167,168).

**ASCOMYCOTA**

*SORADIOMYCETES*

Hypocreales

*Cosmospora viliuscula*

This ascomycete is a nectrioid species having a small, red non-ornamental fruiting body that collapses when dry. The species of Cosmospora are found to grow on polypores and xylariaceous fungi. And Cosmospora viliuscula mainly grows on the xylariaceous fungi (169). Spores are 10 - 10.5 μm in diameter, smooth and ellipsoidal (Fig.4u).

*Xylariales*

*Daldinia eschscholzii*

This tree dwelling fungus is a potential wood decayer. Stomata of the fungi is turbinate to placentiform, sessile, stout stipe, solitary to infrequently aggregated, smooth, 1.5 - 6 cm in diameter and 1 - 4 cm thick. The upper surface is brown to vinaceous, gets blackened and varnished with age. There are dull reddish brown granules found immediately beneath the surface. The fruiting body gets very hard with age. Ascus is 16 – 19.5 cm long and 0.7 – 0.9 cm broad, the spore-bearing part is 7 - 8 cm long with the stipe that is 9 -12 cm long. Ascospores are generally 13-15 µm in diameter, brown to dark brown, unicellular, ellipsoid-inequilateral, with narrowly rounded ends (Fig.4v). It has been reported earlier from Australia, Russia and In Gujarat, India (170,171).

*Hypoxylon rickii*

This sac fungus is found to be genetically similar to *Daldinia*. Ascus of this fungus is discoid and possesses apical rings. The spores are ellipsoidal with a prominent germ slit. The species belonging to the genus *Hypoxylon* are commonly found in the dead wood and has been reported as one of the earliest species to colonize the woods (172).

*Xylaria cirrata*

This fungus is known to have a symbiotic relationship with the termites. This ascomycete is often found growing on the tree stumps in association with the termite nest. The fruiting bodies are white, antler-like and are branched towards the top. The spores of the conidia are asexual. Spores are 4 – 5 μm in diameter, short fusoid to ellipsoid, inequilateral, with narrowly rounded ends that are sometimes pinched, and smooth with a straight germ slit spore length on the less convex side (Fig.4w). It has been reported to be widely present in Taiwan (182).

**References:**

1. Ingold CT. Active liberation of reproductive units in terrestrial fungi. Mycologist; 1999. 113–116 p.

2. Sesartic, Dallafior TN. Global fungal spore emissions, review and synthesis of literature data. Biogeosciences. 2011;8(5):1181–92.

3. Roger Phillips. The mushrooms: Agaricus bohusii [Internet]. RogerMushrooms. 2014 [cited 2015 Jun 22]. p. Rogers Plants Ltd. Available from: http://www.rogersmushrooms.com/gallery/DisplayBlock~bid~12051~gid~~source~gallerydefault.asp

4. Reis FS, Stojković D, Soković M, Glamočlija J, Ćirić A, Barros L, et al. Chemical characterization of Agaricus bohusii, antioxidant potential and antifungal preserving properties when incorporated in cream cheese. Food Res Int [Internet]. 2012;48(2):620–6. Available from: http://linkinghub.elsevier.com/retrieve/pii/S0963996912002050

5. Leonard P. Agaricus dulcidulus [Internet]. Queensland Mycological Society. 2014 [cited 2015 Jun 22]. Available from: http://qldfungi.org.au/resources-2/fungi-keys/fungi-key-agaricus

6. Lenti I. Mushrooms living among us: Mushrooms - world of Batorliget. In: 7th INTERNATIONAL MULTIDISCIPLINARY CONFERENCE. Baia Mare, Romania; 2007. p. 409.

7. F.H. Møller P. Agaricus fuscofibrillosus [Internet]. MycoBank. 1951 [cited 2015 Jun 22]. Available from: http://www.mycobank.org/BioloMICS.aspx?Table=Mycobank&Rec=124146&Fields=All

8. FH.Møller. Danish Psalliota species. Preliminary Studies for a Monograph on the Danish Psalliotae. Part 1. Fresia. 1950;4 (1-2):27.

9. A.Pilát. The Bohemian species of the genus Agaricus. Acta Musei Natl Pragae. 1951;7B:1–19.

10. Arora.D. Mushrooms Demystified: A Comprehensive Guide to the Fleshy Fungi. Berkeley, Calif Ten Speed Press. 1986;326–7.

11. K. Natarajan KN and VK. Basidiocarp production in Agaricus heterocystis Heinem . and Gooss . in nutrient agar media. Curr Sci. 2002;82(9):9–10.

12. Iveiro NN, Lbertó EA. Checklist of the Argentine Agaricales 5. Agaricaceae. Mycotaxon. 2013;(March):1–25.

13. Manimozhi M, Kaviyarasan V. Nutritional Composition and Anti-bacterial Activity Of Indigenous edible mushroom Agaricus Heterocystis. Int J Adv Biotechnol Res. 2013;4(1):78–84.

14. Kuo M. Agaricus hondensis [Internet]. MushroomExpert.Com. 2005 [cited 2015 Jun 22]. Available from: http://www.mushroomexpert.com/agaricus_hondensis.html

15. Lincoff’s GH. Agaricus hondensis (Felt-ringed Agaricus) [Internet]. National Audubon Society Field Guide to North American Mushrooms. 2004 [cited 2015 Jan 1]. Available from: http://www.herbmuseum.ca/content/agaricus-hondensis-felt-ringed-agaricus

16. Murrill WA. The Agaricaceae of the Pacific Coast – III. Brown and black-spored genera. Mycologia. 1912;4 (6):294–308.

17. Terry C. Food habits of three sympatric species of insectovora in western Washington. Can Field-Naturalist. 1978;92:38–44.

18. Jovel E, Kroeger P TN. Hydroquinone: the toxic compound of Agaricus hondensis. Planta Med. 1996;62 (2):195.

19. Phillips R. Mushrooms. Pan MacMillan; 2006.

20. Wasser S. Species nova e genere Agaricus L. ex Fr. emnd. Karst. Nov Sist Nizshikh Rastenii. 1976;13:77–9.

21. Saccardo.P.A. Sylloge Fungorum V: 1007. 1887;XIX: 32(XII):90.

22. Josephine RM, Sahana B. Original Research Article Cultivation of milky mushroom using paddy straw waste. IntJCurrMicrobiolAppSci. 2014;3(12):404–8.

23. D Sreenivasa Murthy ST and NJ. Milky mushroom (Calocybe indica) [Internet]. Indian Institute of Horticultural Research (ICAR). 2012 [cited 2015 Jun 22]. Available from: http://iihr.res.in/content/milky-mushroom-calocybe-indica

24. Vijaykumar G, John P, Ganesh K. Selection of different substrates for the cultivation of milky mushroom. Indian J Tradit Knowl. 2014;13(2):434–6.

25. PM.Kirk. Dictionary of the Fungi. 10th ed. UK: CABI; 2008.

26. Massee GE. Fungi exotici, I". Bulletin of Miscellaneous Informations of the Royal botanical Gardens Kew. 1898. p. 113–36.

27. Kuo M. Chlorophyllum molybdites [Internet]. 2015 [cited 2015 Jun 22]. Available from: http://www.mushroomexpert.com/chlorophyllum_molybdites.html

28. Volk T. Chlorophyllum molybdites, the green spored Lepiota [Internet]. 1999 [cited 2015 Jun 22]. Available from: http://botit.botany.wisc.edu/toms_fungi/aug99.html

29. Beug and Michael W. An Overview of Mushroom Poisonings in North America. The Mycophile. 2004;45(2)(March/April):4–5.

30. Denis RB and. Gastrointestinal syndrome". Mushrooms: poisons and panaceas — a handbook for naturalists, mycologists and physicians. New York: WH Freeman and Company; 1995. 351–377 p.

31. Vellinga EC. Chlorophyllum and Macrolepiota (Agaricaceae) in Australia. Aust Syst Bot. 2003;16(3):361–70.

32. Moreno G, Contu M, Ortega A, Platas G, Peláez F. Molecular phylogenetic studies show Omphalina giovanellae represents a new section of Clitopilus (Agaricomycetes). Mycol Res. 2007;111(12):1399–405.

33. Gyosheva, Melania M.; Denchev CM., Dimitrova EG., Assyov, Boris; Petrova, Roumyana D. & Stoichev GT. Red List of fungi in Bulgaria. Mycol Balc. 2006;3:81–7.

34. Goodwin L. Clitopilus scyphoides var. scyphoides [Internet]. 2013 [cited 2015 Jun 22]. Available from: http://www.leifgoodwin.co.uk/Fungi/Clitopilus scyphoides var. scyphoides.html

35. List BS. Clitopilus scyphoides var. scyphoides [Internet]. Royal Belgian Institute of Natural Sciences. 2016 [cited 2015 Jun 24]. Available from: http://www.species.be/en/601

36. Singer. Clitopilus scyphoides [Internet]. Global Biodiversity Information facility. 2011 [cited 2015 Jun 24]. Available from: http://www.gbif.org/species/3345645

37. Guzmán G, Allen JW, Gartz J. A Worldwide geographical distribution of the Neurotropic fungi, an analysis and discussion. Africa (Lond). 1997;14(1939):1–107.

38. Tóth, Annamária, Hausknecht, Anton, Krisai-Greilhuber, Irmgard, Papp, Tamás, Vágvölgyi, Csaba Vágvölgyi N and LG. Iteratively Refined Guide Trees Help Improving Alignment and Phylogenetic Inference in the Mushroom Family Bolbitiaceae. PLoS One. 2013;8(2).

39. Watling R, Işiloğlu M SH. Observations on the Bolbitiaceae 31. Conocybe volviradicata sp. nov. Mycotaxon. 2010;114:145–9.

40. Malysheva EF. Conocybe mandshurica [Internet]. 2012 [cited 2015 Jun 24]. p. 237. Available from: http://www.mycobank.org/BioloMICS.aspx?Link=T&TableKey=14682616000000063&Rec=53890&Fields=All

41. Japan DDB of. Conocybe mandshurica [Internet]. 2012 [cited 2015 Jun 24]. Available from: http://ddbj.nig.ac.jp/tx_search/search?query=scientific_name_ex:“Conocybe+mandshurica”

42. C.B. Uljé AA and A van I. A new Coprinus from Papua New Guinea sporulating in pure culture. PERS OONIA. 1998;16(Part 4):549–51.

43. Ulje CB& CB. Studies in Coprinus-II. Persoonia. 1991;14:275–339.

44. Kuo M. Coprinellus domesticus [Internet]. MushroomExpert.Com. 2008 [cited 2015 Jun 24]. Available from: http://www.mushroomexpert.com/coprinellus_domesticus.html

45. Emberger G. Coprinellus radians [Internet]. Messiah College. 2008 [cited 2015 Jun 24]. Available from: http://www.messiah.edu/oakes/fungi_on_wood/gilled fungi/species pages/Coprinellus radians.htm

46. Keirle MR, Hemmes DE, Desjardin DE. Agaricales of the Hawaiian Islands . 8 . Agaricaceae : Coprinus and Podaxis ; Psathyrellaceae : Coprinopsis , Coprinellus and Parasola. Fungal Divers. 2004;15:33–124.

47. Secretariat G. Coprinopsis sclerotiorum (Horvers & de Cock) Redhead, Vilgalys & Moncalvo [Internet]. GBIF Backbone Taxonomy. 2013 [cited 2015 Jun 24]. Available from: http://www.gbif.org/species/3332229

48. Smokeylemon. Coprinopsis spelaiophila (Inkcap) [Internet]. T.E.R:R.A.I.N. 2014 [cited 2015 Jun 24]. Available from: http://www.terrain.net.nz/friends-of-te-henui-group/fungi-te-henui/coprinopsis-spelaiophila-inkcap.html

49. Fungorum S. Coprinopsis spelaiophila [Internet]. ZipcodeZoo. 2014. Available from: http://zipcodezoo.com/index.php/Coprinopsis_spelaiophila#Distribution

50. Wood M. Coprinopsis spelaiophila [Internet]. mykoweb. 2013. Available from: http://mushroomobserver.org/name/show_name/15948

51. Buller AH. Coprinus echinosporus. Trans Brit mycol Soc. 1920;6:363.

52. Huijsman. Coprinus giganteoporus. Fungus. 1955;25:19.

53. Redhead V and M. Coprinopsis echinospora. Taxon. 2001;50(1):227.

54. M.Jordan. The Encyclopedia of Fungi of Britain and Europe. David & Charles. 1995. 215 p.

55. Wood W, Smith J WK and LD. Indole and 3-chloroindole: The source of the disagreeable odor of Hygrophorus paupertinus. Mycologia. 2003;95(5):807–8.

56. Clémençon S&. Cystolepiota bucknallii (Berk. & Broome) [Internet]. GSD Species Synonymy. 2014 [cited 2016 Jan 27]. Available from: http://www.speciesfungorum.org/GSD/GSDspecies.asp?RecordID=312540

57. Herrera DM and T. Gymnopilus lepidotus [Internet]. GBIF Data Portal. 2013 [cited 2015 Jun 25]. Available from: http://www.discoverlife.org/mp/20l?id=GBIF370241144

58. Roy Watling, J. C. Frankland, A. M. Ainsworth SI and CHR. Tropical Mycology. CABI; 2002.

59. Gartz J. Occurence of psilocybin, psilocin and baeocystin in Gymnopilus purpuratus. Persoonia. 1989;14:19–22.

60. Davalos LG-. Type studies of Gymnopilus (Agaricales) I. Mycotaxon. 2003;41:43–56.

61. Hoiland K. Gymnopilus purpureosquamulosus and G.ochraceus spp.nov. ( Agaricales, Basidiomycota) - Two new species from Zimbabwe. Mycotaxon. 1998;69:81–5.

62. Secretariat G. Gymnopus gibbosus (Corner) A.W. Wilson, Desjardin & E. Horak [Internet]. GBIF Backbone Taxonomy. 2013. Available from: http://www.gbif.org/species/5449257

63. Wood M. Gymnopus gibbosus (Corner) A.W. Wilson, Desjardin & E. Horak [Internet]. mykoweb. 2010. Available from: http://mushroomobserver.org/name/show_name/21838

64. Kuo M. Gymnopus luxurians [Internet]. MushroomExpert.Com. 2013 [cited 2015 Jun 24]. Available from: http://www.mushroomexpert.com/gymnopus_luxurians.html

65. Murrill. Gymnopus luxurians (Pk.). N Amer Flora. 1916;9:362.

66. Saba, M., Khalid AN. New reports of Gymnopus from Pakistan based on ITS sequences. Mycotaxon. 2014;129(1):63–72.

67. Flower PH& SL. Hymenagaricus (Agaricaceae) de Kerala (Inde) et de Sri Lanka. Bull Jard Bot Nat Belg [Internet]. 2015;54(1):151–82. Available from: http://www.jstor.org/stable/3667871

68. Yang, Z.L.; Ge, Z.W.; Chen C. A new species of the genus Hymenagaricus (Basidiomycota) from Taiwan and its phylogenetic position inferred from ITS and nLSU sequences. Cryptogam Mycol. 2008;29(3):259–65.

69. Sanjuan DW and DT. MushRoaming Colombia: Tropical Rain Forests, Cool Cloud Forests & High Andes [Internet]. Mushroaming Colombia 2015. 2015. Available from: http://mushroaming.com/content/colombia-tour

70. Smith HV. Contributions toward a monograph on the genus Lepiota. I. Type studies in the genus Lepiota. Mycopathol Mycol Appl. 1966;29:97–117.

71. Kumari B, Ns A, Kaur M. Some interesting lepiotoid mushrooms from North India. Mycosphere. 2012;147002:949–55.

72. Garnweidner E. Collins Nature Guide - Mushrooms and Toadstools of Britain and Europe. Collins Nature Guide; 1994.

73. Bartho L. Lepiota castanea [Internet]. Rogers Plants Ltd. 2009 [cited 2015 Jun 26]. Available from: http://www.rogersmushrooms.com/gallery/DisplayBlock~bid~6295~gid~~source~gallerydefault.asp

74. Lezzi T. Lepiota echinacea [Internet]. A.M.I.N.T. 2014 [cited 2015 Jun 26]. Available from: http://www.funghiitaliani.it/index.php?showtopic=27704

75. Paul M. Kirk, Paul F. Cannon DWM and JAS. Dictionary of the Fungi. 10th ed. UK: CABI; 2008.

76. Orton. Lepiota ochraceofulva [Internet]. 1960 [cited 2015 Jun 26]. Available from: http://www.commanster.eu/commanster/Mushrooms/Agaric/SuAgaric/Lepiota.ochraceofulva.html

77. Bessette A. Mushrooms of the Southeastern United States. Syracuse University Press; 2007. 373 p.

78. Clémençon S and. Lepista densifolia (J. Favre). Nov Hedwigia. 1948;23:308.

79. Vesterholt.J KH and. Funga Nordica. 2nd ed. 2012.

80. Peter Dighton J, White JF, Oudemans. The Fungal Community: Its Organization and Role in the Ecosystem [Internet]. CRC Press. 1992. 952 p. Available from: https://www.mendeley.com/research/fungal-community-1/?utm_source=desktop&utm_medium=1.15.1&utm_campaign=open_catalog&userDocumentId={04b568e8-a055-4115-98df-99707b53b0f0}

81. Carlson AL, Justo A, Hibbett DS. Species delimitation in Trametes: a comparison of ITS, RPB1, RPB2 and TEF1 gene phylogenies. Mycologia [Internet]. 2014;106(4):1–11. Available from: http://www.ncbi.nlm.nih.gov/pubmed/24898532

82. Hjelm O. Analysis of halogenated organic compounds in coniferous forest soil from a Lepista nuda (wood blewitt) fairy ring. Chemosphere. 1996;32(9):1719–28.

83. Yafetto L, Carroll L, Cui Y, Davis DJ, Fischer MWF, Henterly AC, et al. The fastest flights in nature: high-speed spore discharge mechanisms among fungi. PLoS One [Internet]. 2008;3(9):e3237. Available from: http://www.pubmedcentral.nih.gov/articlerender.fcgi?artid=2528943&tool=pmcentrez&rendertype=abstract

84. McFarland Joe MG. Edible Wild Mushrooms of Illinois & Surrounding States: A Field-To-Kitchen Guide. University of Illinois Press; 2009. 57–58 p.

85. Zeller SM. New or Noteworthy Agarics from the Pacific Coast States. Mycologia. 1938;30(4):468–74.

86. BCRC. Leucoagaricus bresadolae [Internet]. 2001 [cited 2015 Jun 26]. Available from: http://www.bcrc.firdi.org.tw/fungi/fungal_detail.jsp?id=FU200802120045

87. Bon Schulzer. Leucoagaricus bresadolae. 7th ed. 1977.

88. Uniprot. Leucocoprinus cepistipes [Internet]. 2015 [cited 2015 Jun 25]. Available from: http://www.uniprot.org/taxonomy/876678

89. Mushrooms R. Leucocoprinus cepistipes [Internet]. Rogers Plants Ltd. 2013 [cited 2015 Jun 26]. Available from: http://www.rogersmushrooms.com/gallery/DisplayBlock~bid~6296.asp

90. Roody WC. Mushrooms of West Virginia and the Central Appalachians [Internet]. University Press of Kentucky; 2003. Available from: https://books.google.co.in/books?id=5HGMPEiy4ykC&dq=Leucoagaricus+cepaestipes&source=gbs_navlinks_s

91. Arora D. Mushrooms Demystified. Ten Speed Press. 1986.

92. Mushroom R. Lepiota rubrotincta [Internet]. Rogers Plants Ltd; 2015. Available from: http://www.rogersmushrooms.com/gallery/DisplayBlock~bid~6316.asp

93. Kuo M. Leucoagaricus rubrotinctus [Internet]. MushroomExpert.Com. 2015 [cited 2015 Jun 26]. Available from: http://www.mushroomexpert.com/leucoagaricus_rubrotinctus.html

94. Yuan Y, Li YK, Liang JF. Leucoagaricus tangerinus , a new species with drops from Southern China. Mycol Prog. 2014;13:893–8.

95. Bushra Subair Abdulsada Almaliky,Zainal Abidin Mior Ahmad JK and WM, Yun. Pathogenicity of Marasmiellus palmivorus ( Sharples ) Desjardin comb . Prov . on Oil Palm. Wulfenia. 2012;(June 2015).

96. Huffman J a., Prenni a. J, Demott PJ, Pöhlker C, Mason RH, Robinson NH, et al. High concentrations of biological aerosol particles and ice nuclei during and after rain. Atmos Chem Phys. 2013;13:6151–64.

97. Wannathes.N, Desjardin, D.E., Hyde, K.D., Perry, B.A. and Lumyong S. A monograph of Marasmius ( Basidiomycota ) from Northern Thailand based on morphological and molecular ( ITS sequences ) data. Fungal Divers. 2009;209–310.

98. Antonín V, Buyck B. Marasmius (Basidiomycota, Marasmiaceae) in Madagascar and the Mascarenes. Fungal Divers [Internet]. 2006;23(1908):17–50. Available from: http://www.scopus.com/inward/record.url?eid=2-s2.0-33846559869&partnerID=tZOtx3y1

99. Berch R. Standardized Inventory Methodologies for Components of British Columbia’s Biodiversity: Macrofungi (Including the Phyla Ascomycota and Basidiomycota). In: Version 1. Province of British Columbia: Resources Inventory Committee.; 1997. Available from: https://www.for.gov.bc.ca/hts/risc/pubs/tebiodiv/macrofungi/

100. Kuo M. Marasmius nigrodiscus [Internet]. MushroomExpert.Com. 2012 [cited 2015 Jun 29]. Available from: http://www.mushroomexpert.com/marasmius_nigrodiscus.html

101. Walter J. Sundberg And MFD. The Taxonomy, Ecology, And Distribution of Marasmius ( Agaricales, Tricholomataceae) In Illinois. Trans Illinois Acad Sci. 1989;82(3):109–20.

102. Wannathes N, Desjardin DE, Lumyong S. Mating studies, new species, and new reports of Marasmius from northern Thailand. Mycol Res. 2007;111(8):985–96.

103. Desjardin DE. The Genus Marasmius from the Southern Appalachian Mountains [Internet]. The University of Tennessee, Knoxville; 1989. Available from: http://trace.tennessee.edu/utk_graddiss/2513

104. Kiyashko A, Malysheva E. Fungi of the Russian Far East 2. New species and new records of Marasmius and Cryptomarasmius (Basidiomycota). Phytotaxa [Internet]. 2014;(June 2015). Available from: http://www.researchgate.net/profile/Ekaterina_Malysheva/publication/268506841_Fungi_of_the_Russian_Far_East_2._New_species_and_new_records_of_Marasmius_and_Cryptomarasmius_(Basidiomycota)/links/54789f840cf293e2da2b2914.pdf

105. Volk TJ. Marasmius oreades, the fairy ring mushroom [Internet]. Tom Volk’s Fungus of the Month for March 2003. 2003 [cited 2015 Jun 29]. Available from: http://botit.botany.wisc.edu/toms_fungi/mar2003.html

106. Kuo M. Marasmius oreades [Internet]. MushroomExpert.Com. 2013. Available from: http://www.mushroomexpert.com/marasmius_oreades.html

107. Zhao R, Desjardin DE, Soytong K, Perry BA, Hyde KD. A monograph of Micropsalliota in Northern Thailand based on morphological and molecular data. Fungal Divers [Internet]. 2010;45(1):33–79. Available from: http://www.springerlink.com/content/p407tv78828w4r24/

108. Ka, K.H.; Park, H.; Hur, T.C.; BAc W. Formation of fruiting body of Omphalotus japonicus by sawdust cultivation. Korean J Mycol. 2010;38(1):80–82.

109. Kirchmair.M, S. Morandell DS and RP. Phylogeny of the Genus Omphalotus Based on Nuclear Ribosomal DNA-Sequences. Mycologia. 2004;96(6):1253–60.

110. Kirchmair M, Morandell S, Stolz D, Pöder R SC. Phylogeny of the genus Omphalotus based on nuclear ribosomal DNA-sequences. Mycologia. 2004;96(6):1253–60.

111. Moreno, G.; Esteve-Raventós, F.; Pöder, R.; Ayala N. Omphalotus olivascens var. indigo, var.nov. from Baja California (Mexico). Mycotaxon. 1993;48:217–22.

112. Ammirati, J.F., J.A. Traquair PAH. Poisonous Mushrooms of the Northern United States and Canada. Minneapolis: University of Minnesota Press; 1985.

113. Alfredo Vizzini and Enrico Enrole. Paralepistopsis gen. nov. and Paralepista (Basidiomycota, Agaricales). Mycotaxon. 2014;120:253–67.

114. Redhead V and H. Parasola auricoma (Pat.) [Internet]. MycoBank. 2001 [cited 2015 May 2]. Available from: http://www.mycobank.org/name/Parasola auricoma

115. Hesler AHS& LR. The North American Species of Pholiota [Internet]. MykoWeb. 1951. Available from: http://www.mykoweb.com/Pholiota/species/Pholiota_spumosa.html

116. Srivastava M. A pink colour Pleurotus djamor (Rumph.) Boedijn from natural habitat of north Bihar, India. Curr Sci. 2001;80(3):337–8.

117. Banerjee, P. and Sundberg WJ. The Genus Pluteus Section Pluteus (Pluteaceae, Agaricales) in the Midwestern United States. Mycotaxon. 1995;53:189–246.

118. Singer R. Contributions Towards a Monograph of the Genus Pluteus. Trans Br Mycol Soc 39(2) 145-232. 1956;39(2):145–232.

119. Guzmán G, Allen JW, Gartz J. A Worldwide geographical distribution of the Neurotropic fungi, an analysis and discussion. Ann Mus.civRovereto. 1997;14(1939):1–107.

120. Frank JL, Coffan RA, Southworth D., JL, Coffan, RA and Southworth D. Aquatic gilled mushrooms: Psathyrella fruiting in the Rogue River in southern Oregon. Mycologia. 2010;102(1):93–107.

121. Smith AH. The North American Species of Psathyrella. The New York Botanical Garden Press; 1972. 633 p.

122. Gibson I. Psathyrella in the Pacific Northwest [Internet]. South Vancouver Island Mycological Society. 2007 [cited 2015 May 4]. Available from: http://www.svims.ca/council/Psathy.htm

123. Robin A Ohm, De Jong. JF, Lugones. LG, Aerts. A, Kothe. E, Stajich. JE, De Vries. RP et al. "Genome sequence of the model mushroom Schizophyllum commune. Nat Biotechnol. 2010;28(9):957–63.

124. Arun Kumar Dutta, Prakash Pradhan AR and KA. Agaricales of West Bengal, India. I. Clavariaceae: Clavaria and Scytinopogon. Indian J Appl Pure Bio. 2012;27:53–8.

125. Natarajan K. South Indian Agaricales V: Termitomyces heimii. Mycologia. 1979;61(2):170–4.

126. Karun NC, Sridhar KR. Occurrence and distribution of Termitomyces ( Basidiomycota , Agaricales ) in the Western Ghats and on the west coast of India. Czech Mycol. 2013;65(2):233–54.

127. Yu X, Deng H, Yao Y. Leucocalocybe, a new genus for Tricholoma ongolicum (Agaricales, Basidiomycota). African J Microbiol Res [Internet]. 2011;5(31):5750–6. Available from: http://www.academicjournals.org/AJMR/abstracts/abstracts/abstract 2011/23Dec/Yu et al.htm

128. Castro AJ and ML. The genus Volvariella in Spain: V. dunensis comb. & stat. nov. and observations on V. earlei. Mycotaxon. 2010;112(June):261–70.

129. Kaur NJ SM and KH. Two new species of agarics from India. Mycosphere. 2013;4(4):856–63.

130. Butler E. Trial field key to the species of VOLVARIELLA in the Pacific Northwest [Internet]. Snohomish County Mycological Society. 2012 [cited 2015 May 4]. Available from: http://www.svims.ca/council/Volvar.htm

131. Jia B-S, Zhou L-W, Cui B-K, Rivoire B, Dai Y-C. Taxonomy and phylogeny of *Ceriporia* (Polyporales, Basidiomycota) with an emphasis of Chinese collections. Mycol Prog [Internet]. 2013;13(1):81–93. Available from: http://link.springer.com/10.1007/s11557-013-0895-5

132. Wang J, Yao L-Y, Lu Y. Ceriporia lacerata DMC1106, a new endophytic fungus: Isolation, identification, and optimal medium for 2′,4′-dihydroxy-6′-methoxy-3′,5′-dimethylchalcone production. Biotechnol Bioprocess Eng [Internet]. 2013;18(4):669–78. Available from: http://link.springer.com/10.1007/s12257-012-0846-z

133. Marcinkevičienė L, Vidžiūnaitė R, Tauraitė D, Rutkienė R, Bachmatova I, Morkūnas M, et al. Characterization of laccase from Coriolopsis byrsina GRB13 and application of the enzyme for synthesis of redox mediators. Chemija. 2013;24(1):48–58.

134. Kuo M. Coriolopsis gallica [Internet]. MushroomExpert.Com. 2010 [cited 2015 May 4]. Available from: http://www.mushroomexpert.com/coriolopsis_gallica.html

135. Gilbertson, R. L. and LR. North American Polypores, Volume 1. Oslo: Fungiflora; 1986. 1-433 p.

136. Ryv. G and. Earliella scabrosa (Pers.). Mycotaxon. 1985;22:364.

137. Roy ABD and A. Studies on Indian Polypores. IV.Morphlogical and cultural characters of Polyporus grammocephalus. Mycologia. 1981;73(1):150–6.

138. Mtui G, Nakamura Y. Lignocellulosic enzymes from Flavodon flavus, a fungus isolated from Western Indian Ocean off the coast of Dar es Salaam, Tanzania. African J Biotechnol. 2008;7(17):3066–72.

139. Kuo M. Fomes fomentarius [Internet]. MushroomExpert.Com. 2010 [cited 2015 May 10]. Available from: http://www.mushroomexpert.com/fomes_fomentarius.html

140. Kuo M. Ganoderma applanatum [Internet]. MushroomExpert.Com. 2004 [cited 2015 May 24]. Available from: http://www.mushroomexpert.com/ganoderma_applanatum.html

141. Roberts BSP. Fungi. Collins; 2005. 126 p.

142. Rogers Mushroom. Ganoderma carnosum [Internet]. Rogers Plants Ltd. 2012 [cited 2015 May 24]. Available from: http://www.rogersmushrooms.com/gallery/DisplayBlock~bid~12133~gid~~source~gallerydefault.asp

143. Volk KE and T. Ganoderma lucidum, Reishi or Ling Zhi, a fungus used in oriental medicine [Internet]. Tom Volk’s Fungus of the Month for March 2005. 2005 [cited 2015 May 25]. Available from: http://botit.botany.wisc.edu/toms_fungi/mar2005.html

144. Socala K. Evaluation of Anticonvulsant, Antidepressant-, and Anxiolytic-like Effects of an Aqueous Extract from Cultured Mycelia of the Lingzhi or Reishi Medicinal Mushroom Ganoderma lucidum (Higher Basidiomycetes) in Mice. Int J Med Mushrooms. 2015;17(3):209–18.

145. Taiwan B. Ganoderma multipileum - Lingzhi [Internet]. Taiwan Biota Project. 2014 [cited 2015 May 25]. Available from: http://biotataiwan.org/Ganoderma-multipileum.html

146. Ubaidillah NHN, Abdullah N, Sabaratnam V. Isolation of the intracellular and extracellular polysaccharides of Ganoderma neojaponicum (Imazeki) and characterization of their immunomodulatory properties. Electron J Biotechnol [Internet]. 2015;18(3):188–95. Available from: http://www.sciencedirect.com/science/article/pii/S0717345815000421

147. Kuo M. Ganoderma tsugae [Internet]. MushroomExpert.Com. 2004 [cited 2015 Apr 7]. Available from: http://www.mushroomexpert.com/ganoderma_tsugae.html

148. Mizuno T, Wang G ZJ et al. Reishi, Ganoderma lucidum and Ganoderma tsugae: bioactive substances and medicinal effects. Food Rev Intl. 1995;11(1):151–66.

149. Grand BC and LF. Lenzites elegans (Spreng.) Pat. Mycological Herbarium NCSU; 2011. p. 9–11.

150. Nakasone KK. Cultural studies and identification of wood-inhabiting Corticiaceae and selected Hymenomycetes from North America. Mycol Mem. 1990;15(1):1–412.

151. Chamuris G. The non-stipitate steroid fungi in the northeastern United States and adjacent Canada. Mycol Mem. 1988;14:1–247.

152. Cooper J. Materials for a Checklist of Pacific Island Basidiomycetes ( excluding Rusts and Smuts ). Landcare Res. 2011;(July):1–189.

153. Szczepkowski A. Perenniporia Fraxinea ( Fungi, Polyporales), a New Species for Poland. Polish Bot J. 2004;49(1):73–7.

154. Bao-kai CUI, Henning DAIY, Ecology A. Two species of Hymenochaetaceae ( Basidiomycota , Aphyllophorales ) new to China. Fung Sci. 2007;2:130117–8.

155. Olusegun O V. Molecular Identification of Trametes Species Collected from. Jordan J Biol Sci. 2014;7(3):165–9.

156. Desertas S. The Fungi Which Cause Plant Diseases [Internet]. Scribd. 2011 [cited 2015 May 25]. Available from: https://www.scribd.com/doc/72545650/6/INDEX

157. Knezević A, Milovanović I, Stajić M, Vukojević J. Trametes suaveolens as ligninolytic enzyme producer. Zb Matice Srp za Prir Nauk / Matica Srp Proc Nat Sci [Internet]. 2013;(124):437–44. Available from: http://www.doiserbia.nb.rs/Article.aspx?ID=0352-49061324437K

158. Cao Y, Yuan HS. Ganoderma mutabile sp nov from southwestern China based on morphological and molecular data. Mycol Prog [Internet]. 2013;12(1):121–6. Available from: <Go to ISI>://WOS:000313659700009

159. Erlon. Truncospora ochroleuca (Berk.) Pilát [Internet]. Mushroomobserver.org. 2015 [cited 2015 May 25]. Available from: http://mushroomobserver.org/name/show_name/49141

160. Ediriweera S. A new record of Fulvifomes fastuosus from Sri Lanka. J Natl … [Internet]. 2014;42(4):369–71. Available from: http://www.sljol.info/index.php/JNSFSL/article/view/7737

161. Herrera B and S. Fulvifomes fastuosus (Lév.). Mikol i Fitopatol. 1992;26(1):13.

162. Forest B, Service F, Gilbertson RL. Studies of two species of Phellinus in western north america. Mycopathol Mycol Appl. 1972;46(4):351–65.

163. Ryvarden. Pyrrhoderma scaurum (Lloyd). Mycotaxon. 1990;38:97.

164. Anderson R. Geastrum pectinatum Pers. (Gasteromycetes: Lycoperdales), an Earth Star New to Ireland. Irish Nat J [Internet]. 2015;24(9):357–60. Available from: http://www.jstor.org/stable/25539887

165. Ellis J B EMB. Fungi without Gills (Hymenomycetes and Gasteromycetes): an Identification Handbook. London: Chapman and Hall; 1990.

166. Rogers Mushroom. Geastrum striatum [Internet]. Rogers Plants Ltd. 2014 [cited 2015 May 27]. Available from: http://www.rogersmushrooms.com/gallery/DisplayBlock~bid~12136~gid~~source~gallerydefault.asp

167. Mohanan C. Macrofungal diversity in the Western Ghats , Kerala , India : members of Russulaceae. J Threat Taxa. 2014;6(April):5636–48.

168. Mohanan.C. Macrofungi of Kerala. Kerala Forest Research Institute; 2011. 597 p.

169. Cesar S Herrera, Amy Y Rossman, Gary J Samuels, Olinto Liparini Pereira PC. Systematics of the Cosmospora viliuscula species complex. Mycologia. 2015;107(3):532–57.

170. DeShazer D. Daldinia eschscholzii [Internet]. Taiwan Biodiversity National Information Network. 2008 [cited 2015 May 27]. Available from: http://www.discoverlife.org/mp/20q?search=Daldinia+eschscholzii&b=EOL/pages/150938

171. Rehm. Daldinia eschscholtzii (Ehrenb.). Ann Mycol. 1904;2(2):175.

172. Huei-Mei H, Yu-Ming J, Rogers JD. Molecular phylogeny of Hypoxylon and closely related genera. Mycologia. 2005;97(4):844–865.
